# POZ/BTB and AT hook containing zinc finger 1 (PATZ1) suppresses differentiation and regulates metabolism in human embryonic stem cells

**DOI:** 10.7150/ijbs.83927

**Published:** 2024-01-21

**Authors:** Min Huang, Xiaohua Liao, Xuepeng Wang, Yiwei Qian, Wensheng Zhang, Guokai Chen, Qiang Wu

**Affiliations:** 1 The State Key Laboratory of Quality Research in Chinese Medicine, Macau University of Science and Technology, Macao SAR 999078, China.; 2 Cam-Su Genomic Resource Center, Medical College of Soochow University, Suzhou 215123, China.; 3 Faculty of Health Sciences, University of Macau, Taipa, Macao SAR 999078, China.; 4 The Precision Regenerative Medicine Research Centre, Macau University of Science and Technology, Taipa, Macao SAR 999078, China.

**Keywords:** PATZ1, human embryonic stem cells (hESCs), pluripotency, mitochondrial function, glycolysis

## Abstract

Human embryonic stem cells (hESCs) can proliferate infinitely (self-renewal) and give rise to almost all types of somatic cells (pluripotency). Hence, understanding the molecular mechanism of pluripotency regulation is important for applications of hESCs in regenerative medicine. Here we report that PATZ1 is a key factor that regulates pluripotency and metabolism in hESCs. We found that depletion of PATZ1 is associated with rapid downregulation of master pluripotency genes and prominent deceleration of cell growth. We also revealed that PATZ1 regulates hESC pluripotency though binding the regulatory regions of *OCT4* and *NANOG*. In addition, we demonstrated PATZ1 is a key node in the OCT4/NANOG transcriptional network. We further revealed that PATZ1 is essential for cell growth in hESCs. Importantly, we discovered that depletion of PATZ1 drives hESCs to exploit glycolysis which energetically compensates for the mitochondrial dysfunction. Overall, our study establishes the fundamental role of PATZ1 in regulating pluripotency in hESCs. Moreover, PATZ1 is essential for maintaining a steady metabolic homeostasis to refine the stemness of hESCs.

## Introduction

Human embryonic stem cells (hESCs) derived from inner cell mass of blastocyst-stage embryos can proliferate infinitely (self-renewal) and differentiate into all cell types of an adult organism (pluripotency) 1-3. Due to their self-renewal and pluripotency, hESCs have great clinical potential in cell replacement therapies. The pluripotency state is maintained by expression of pluripotency genes while repression of developmental genes 4. Master pluripotency regulators, OCT4, NANOG and SOX2 establish an extensive self-regulatory loop circuity through regulating their promoters 5. Recent studies discover that many other pluripotency regulators refine the transcriptional circuity in ESCs 6-8. Thus, identifying novel unknown pluripotency regulators is helpful to understand the gene regulatory network that controls hESC identity.

hESCs present a unique metabolic program in terms of maintaining their pluripotency. A metabolic switch from glycolysis to mitochondrial oxidative phosphorylation (OXPHOS) for sufficient ATP production is required for ESCs to differentiate. Distinct from somatic cells, pluripotent stem cells are prone to exploit glycolysis because vast glucose molecules are recruited into the glycolytic flux to support robust ES cell growth 8-10. Indeed, undifferentiated hESCs display strong stemness under physiological oxygen level in which OCT4, NANOG and SOX2 are highly expressed 11-13. Although glycolysis offers substantial energy production to hESCs, mitochondrial OXPHOS is a still indispensable metabolic process for sustaining cell survival and differentiation 14. During differentiation, hESCs increase the number of mitochondria and create mature morphology 15, 16, which is caused by oxygen consumption and mitochondria respiration 11. However, excessive mitochondrial respiration may induce DNA damage and epigenetic alteration. Therefore, hESCs need to tightly regulate the balance of glycolysis and OXPHOS.

POZ/BTB and AT hook containing zinc finger 1 (PATZ1), also known as ZNF278, is a transcriptional factor which may positively or negatively regulate gene transcription subjected to the cellular contents 17, 18. PATZ1 structurally contains a BTB/POZ domain at the N-terminal, two AT-hook domains in the central region, and serval C2H2-ZF motifs at the C-terminal 19. Of note, the AT-hook domains of PATZ1 provide a site of undergoing chromatin remodeling, in which proteins bind to DNA during transcription 20. PATZ1 may have high affinity to GC-rich DNA regions by configurating with its zinc fingers domain 21. Indeed, our lab have previously identified that the Patz1 has AT-rich or GC-rich binding motif due to its multiple domains 22. The diversity of domains allows PATZ1 to play multiple roles in gene regulation. Tumor suppressive role of PATZ1 has been found in thyroid cancer cells 23-25, testicular germ cells 26 and liver cancer cells 27. Conversely, PATZ1 may act as an oncogene because it promotes the cell cycle in colon cancer 28. In mouse embryonic development, homozygous *Patz1*-knockout mice leaded to CNS developmental defects and probably result in perinatal death due to malformations in the in ventricular outflow tract 29. Deletion of *Patz1* in mouse embryonic fibroblasts declines the cell proliferation through blocking p53 to bind to its response elements 30. In mouse ESCs, deletion of *Patz1* activates global differentiation and abolishes ES cell pluripotency 22. Patz1 modulates the iPSCs reprogramming process in a dosage-dependent manner 31. Furthermore, PATZ1 is essential for neural stem cell maintenance and proliferation 32.

To date, limited research in PATZ1 has been conducted in hESCs. In this study, we identify that PATZ1 is an essential pluripotency regulator in hESCs. We have systematically studied the necessity of PATZ1 in hESC pluripotency and metabolic homeostasis.

## Materials and Methods

### Cell culture

H1 hESCs (WA01) were maintained in TeSR-E8 medium (STEMCELL Technologies) on 0.5 mg/6-well plate of Matrigel (BD Bioscience) coated tissue-culture plates with daily medium feeding 33. Cells were passaged every 2 to 3 days with 0.5 mM EDTA (Gibco) in Dulbecco's phosphate-buffered saline (Gibco) at 1:6 to 1:12 ratio.

For EB differentiation, hESCs were hanged with density of 1000 cells/20 ul/drop for first two days with Poly (vinyl alcohol) (Sigma) 34, and subsequently transferred to a ultra-low attachment surface plate (Corning) for additional six days. The cells were fed with differentiation medium containing KnockOut® DMEM (Gibco) supplemented with 20% KnockOut® Serum Replacement (Gibco), 1% nonessential amino acids (Gibco), L-glutamine (Gibco), and beta-mercaptoethanol (Gibco). On Day 8, we subsequently plated suspended EB cells onto a 0.1% gelatin-coated (Gibco) culture dish (NEST Biotechnology) directly and fed with DMEM/High Glucose (Gibco) supplemented with 10% fetal bovine serum (FBS) (Gibco) for further 6 days. The morphology of EB cells was monitored by an inverted phase microscopy (Leica). EB cells were harvested every two days for down-stream analysis.

HEK 293T cells (ATCC) were cultured in medium consisting of DMEM/High Glucose (Gibco) supplemented with 10% fetal bovine serum (Gibco). All cells were cultured at 37°C in a humidified atmosphere containing 5% CO_2_.

### Construction of plasmids, lentiviral packaging, stable cell line generation and dual-luciferase assays

For knockdown cell lines, shRNA specifically targeting human *PATZ1* gene were designed and cloned into pSUPER RNAi vector (OligoEngine). The two most efficient sequences were cloned in a lentiviral vector of pPLK_GFP_Puro (PPL) to establish stable knockdown cell lines. In brief, lentiviral vectors were packed by co-transfecting TAT, REV, VSVG and GAG (Addgene) into HEK293T cells, concentrated and stored at -80℃ 35. Cells were infected with viral particles in culture medium containing 1 µg/ml polybene (Solarbio). After 1 µg/ml puromycin (Solarbio) selection for 7 days, GFP-positive single clones were picked and expanded. The efficiency of knockdown was determined by RT-qPCR and confirmed by western blotting.

For plasmids used in luciferase assays, *OCT4* CR2, CR3 and CR4 regions was amplified by PCR method and cloned into the pGL3-Promoter vector (Promega) upstream of the *firefly luciferase* gene to generate the *OCT4* CR2-pSV40-Luc, *OCT4* CR3-pSV40-Luc, *OCT4* CR4-pSV40-Luc luciferase reporter plasmids. *NANOG* proximal promoter was amplified by PCR method and cloned into the pGL3-Basic vector (Promega) to produce the p*NANOG* PP-*Luc* plasmid. Cells were transfected by negative control- and *PATZ1*- shRNA (2 µg) for 48 hours and followed puromycin selection. The puromycin-resistant cells were transfected with luciferase reporters (600 ng), and an internal control pRL-TK (30 ng) encoding *Renilla luciferase*.* Firefly* and *Renilla luciferase* activities were measured with the TransDetect^®^ Double-Luciferase Reporter Assay Kit (TransGen) according to the manufacturer's instructions. The results generated from the RNAi-treated cells were compared with the cell transfected with empty vector. The *firefly* data was normalized to the *Renillia luciferase* readings.

PCR primers for plasmid construction were available in the Supplementary Table 1.

### Colony formation assay, alkaline phosphatase staining, Wright-Giemsa staining and JC-1 staining

For colony formation, cells were transfected with negative control- and *PATZ1*-shRNA for 6 hours. The transfected cells were harvested and re-seeded at a density of 1000 cells per well in a 6-well plate. Cells were stained with 0.2% w/v crystal violet (Solarbio) 15 days after re-seeding. Cells were washed three times after the crystal violet solution being discarded. The plates were air-dried, and the visible colonies were photographed.

Alkaline phosphatase staining was performed using Alkaline Phosphatase Detection Kit (Biyuntian) according to manufacturer's instructions. The images were captured using the Leica DFC310 FX light microscope system (Leica).

Wright-Giemsa staining was performed using Modified Giemsa Staining Solution (Biyuntian) according to the manufacturer's instruction. The images were captured using the Leica DFC310 FX light microscope system (Leica).

For JC-1 staining, cells were transfected with negative control- and *PATZ1*-shRNA for 6 hours. The transfected cells were harvested and re-seeded at a density of 5000 cells per 35 mm dish (NEST Biotechnology). Freshly prepared media were added to the samples, and 10 µg/ml of JC-1 (Biyuntian) solution was added 36. After 30 min of incubation, cells were immediately analyzed for JC-1 by confocal laser microscope system (Leica).

### Nuclear/cytoplasmic protein extraction, Immunostaining, Co-IP, Western blotting, and RT-PCR

Nuclear/cytoplasmic protein extraction was performed using ProteinExt^®^ Mammalian Nuclear and Cytoplasmic Protein Extraction Kit (TransGen Biotech) according to manufacturer's instructions. Protein fractions were analyzed by western blotting.

For immunostaining, cells were fixed in 4% paraformaldehyde solution (Solarbio) and permeabilized by PBS containing 0.1% Triton X-100 (Solarbio). After incubation with 4% FBS/PBS, cells were incubated with primary antibodies followed by fluorescence secondary antibodies (Abcam). Nuclei were stained with 1 μg/ml DAPI (Solarbio). Images were captured using a confocal laser microscope system (Leica).

For Co-IP, endogenous proteins were lysed by NP40 buffer (Cell Signaling Technology) containing protease inhibitor cocktail (Roche). After centrifugation, the protein fraction was incubated with Dynabeads (Invitrogen) coupled primary antibodies over night at 4℃. After washing, the bound complexes were eluted by boiling for 10 min in 2x SDS loading buffer (Cell Signaling Technology) and analyzed by western blotting assay.

For western blotting analysis, cells were lysed in RIPA buffer (Cell Signaling Technology) containing protease inhibitor cocktail (Roche). Proteins were separated by SDS-PAGE and transferred onto a PVDF membrane (Millipore). After blocking with non-fat dry milk (Bio-Rad), the membrane was incubated with primary antibodies followed by secondary antibodies (Santa Cruz) and visualized with UltraSignal ECL Western Blotting Detection Reagent (4A Biotech).

For RT-PCR, total RNA was isolated using TRIzol (Invitrogen) and purified by RNeasy kit (Qiagen) according to the manufactures' protocol. cDNA was synthesized using NovoScript® Ⅲ Reverse Transcriptase kit (Invitrogen) with oligo(dT) primers (Novoprotein). Quantitative PCR was performed using 2×SYBRGreen qPCR Mix (Goyoobio) on ViiA 7 Real-Time PCR System (Applied Biosystems). The relative quantification of mRNA levels was computed using 2^-ΔΔCT^ method. *β-Actin* was used as an endogenous control.

RT-PCR primers and primary antibodies used were available in the Supplementary Table 2 and Supplementary Table 3 respectively.

### Flow cytometry

Carboxy-fluorescein diacetate succinimidyl ester (CSFE) (Invitrogen) incorporation was used to uncover the proliferative status and cell cycle duration of hESCs, and was performed as previously described 37. Cells were incubated with 3 mg/ml CFSE in PBS for 15 min at 37℃ and unincorporated CFSE was removed through several washes with DMEM/F12 (Gibco) supplemented with 20% KnockOut® Serum Replacement (Gibco), which contained excess protein to quench unbound CFSE. Cells were seeded in a density of 2× 10^5^ cells in 6-well plates. On the next day, the cells were transfected by negative control- and *PATZ1*- shRNA as described above. Samples were harvested after 48 hours starting from the onset of treatment and prepared for autofluorescent detection of CFSE incorporation using FACScan flow cytometry (Becton Dickinson).

For cell cycle analysis, cells were seeded and transfected with the negative- and *PATZ1*- shRNA for 6 hours. After 48 hours of transfection, cells were harvested and re-suspended in cold PBS. The cells were dropwise fixed in cold 70% ethanol and incubated in -20℃ overnight. Cells were stained with the Cell Cycle and Apoptosis Kit (US EVERBRIGHT) according to the manufacturer's protocol. After 30 minutes of incubation, the cells were recovered by gentle centrifugation. The cell pellet was washed with PBS twice. Cells were immediately analyzed for cell cycle assay by FACScan flow cytometry (Becton Dickinson).

For cell apoptosis analysis, cells were transfected by negative control- and *PATZ1*- shRNA for 48 hours. Cells were collected and stained with the Annexin V-FITC Apoptosis Detection Kit (Acmec) according to the manufacturer's protocol. After 30 min of incubation, apoptosis of the cells was immediately analyzed by FACScan flow cytometry (Becton Dickinson).

For TMRM measurement, cells were transfected by negative control- and *PATZ1*- shRNA for 48 hours. Cells were incubated with culture medium supplemented with 10 μM TMRM (Invitrogen). After 30 min of incubation, TMRM of cells was immediately analyzed by FACScan flow cytometry (Becton Dickinson). All flow cytometry loaded an unstained fluorescence sample as negative controls. All flow cytometry results were analyzed by FlowJo (Becton Dickinson).

### Chromatin Immunoprecipitation (ChIP) assay, ChIP-qPCR and ChIP-sequencing

ChIP was performed as previously described 38. In brief, wild-type H1 hESCs were cross-linked with 1% formaldehyde (Sigma) for 10 min prior to neutralization with 0.2 M glycine (Sigma) for 5 min at room temperature. Cells were pelleted and washed with cold PBS. The nuclei were lysed in lysis buffer containing 1% SDS (Sigma), followed by sonication. Sonicated chromatin was immunoprecipitated with Protein G Dynabeads (Invitrogen) coated with anti-PATZ1 (Santa Cruz). The beads were washed and incubated for 45 min at 68℃ with agitating at 1400 rpm. The eluent was de-crosslinked by pronase (Invitrogen). DNA was precipitated and dissolved in nuclease-free water (Invitrogen) for real-time PCR. For ChIP-qPCR, enrichment folds were calculated by determining the apparent IP efficiency (ratios of the amount of ChIP enriched DNA over that of the input sample) and normalized to the level observed at a control region.

For ChIP-sequencing (ChIP-seq), ChIP-DNA library was prepared by TransNGS^®^ DNA Library Prep Kit for Illumina^®^ (TransGen). High-throughput sequencing was then performed with the Illumina Novaseq 6000 (Illumina). ChIP-Seq data was processed and analyzed using the CSI NGS portal (https://csibioinfo.nus.edu.sg/csingsportal) 39. In brief, raw fq.gz files were mapped to the GRCh38 (hg38) human genome assembly using bowtie2 40. The read coverage was normalized according to the sequencing depth. Peak calling and annotation were performed by comparing with the input file and pointed out by MACS2 with acceptable model fold and q-Value 41. The enriched motifs were discovered by HOMER 42.

For location classification, ChIP-seq peaks were annotated by mapping the locations of all transcriptional starting sites (TSS) and transcriptional terminal sites (TTS) in human genome with Perl script. Locations of the genome were defined in accordance with the position and length. 10 - 1 kb upstream of the TSS defined as upstream. 1 kb upstream of the TSS to the TSS defined as TSS. The region between TSS and TTS was defined as gene body. 1 kb downstream of the TTS and to the TTS den fined as TTS. 10 - 1 kb downstream of the TTS defined as downstream. Top 100-ranked peak heights of PATZ1-ChIP peaks were available in the Supplementary Table 4.

### RNA-seq analysis

For RNA-seq, RNA was isolated and purified using FastPure^®^ Cell/Tissue Total RNA Isolation Kit V2 (Vazyme). Strand-specific RNA-seq libraries were prepared using TransNGS^®^ RNA-Seq Library Prep Kit for Illumina^®^ (TransGen). Samples were sequenced on the Illumina Novaseq 6000. platform (Illumina), rendering 100-bp paired-end reads. RNA-Seq data was processed and analyzed for differential expression using the CSI NGS portal (https://csibioinfo.nus.edu.sg/csingsportal) 39. All peaks in PATZ1 RNA-seq were available in the Supplementary Table 5.

### Mitochondrial respiration measurements

For mitochondrial respiration measurements, stable cell lines with lentiviral *PATZ1* knockdown were used. The oxygen-consumption rate (OCR) and extracellular acidification rate (ECAR) of H1 hESCs were measured by Seahorse XF Extracellular Flux Analyzer (Agilent) and was performed according to the manufacturer's protocol. Briefly, 20,000 hESCs/well were seeded onto a Matrigel^®^-coated XF96 cell culture microplate (Agilent) and cultured overnight in 80 μl of hESC culture medium. One hour before the assay culture medium was changed to pH 7.4 XF assay medium supplemented with 1 mM pyruvate (Gibco), 2 mM glutamine (Gibco), and 10 mM glucose (Sigma), and incubated at the incubator without supplied CO_2_ for 1 h before the completion of probe cartridge calibration. Basal respiration was measured in the XF assay medium without oligomycin, and mitochondrial function was measured by injecting 2 μM oligomycin (Sigma), 2 μM FCCP (Sigma), and 1 μM rotenone (Sigma) mix with 1 μM antimycin A (Sigma) for OCR assays and 10 mM glucose, 1 mM oligomycin, and 50 mM 2-DG (Sigma) for ECAR assays. After the test, the total protein in each well was measured by SRB (Sigma) method and the data were normalized on proteins. OCAR and ECAR results were analyzed using the calculation method described by Mookerjee et al. 43, 44. Equations were available in the Supplementary Table 6.

### Metabolome analysis

For metabolome analysis, stable cell lines with lentiviral *PATZ1* knockdown were used. Cells well harvested at density of 1× 10^6^ cells and sonicated with 1ml of chilled 80% methanol with internal standards. The samples were centrifuged at 18000 g for 14 min at 4℃. Exact 40 μL of supernatant were transferred to a 96 well microplate. Each well was treated with 20 μL of 200 mM 3-NPH and 20 μL of 120 mM EDC for 60 min at 30℃ with agitation of 1450 rpm. The mixtures were diluted with chilled methanol and followed by centrifuged at 4000 g for 20 min at 4℃. 150 μL of supernatant were transferred in a clean vial for injection.

The Acquity-I Xevo TQ-S LC/MS instrument (Waters) was used for the detection of energy metabolism-related metabolites. The instrument was routinely maintained every 48 hours. The UPLCMS/MS parameters were set as: Column: BEH C18 1.7 µM analytical column (2.1 × 100 mm); Column temperature: 40℃; Sample temperature: 10℃; mobile phase: A=5mM DIPEA aqueous solution, B= ACN: IPA=7: 3; Elution conditions: 0-1 min (1% B), 1-9.5 min (1-15% B), 9.5-13 min (15-62% B), 13-14min (62-100% B), 14-16 min (100% B), 16-16.2 min (100-1% B), 16.2-18 min (1% B); Flow rate: 0.3 mL/min; Injection volume: 5.0 µL. The mass spectrometer parameters were set as: Capillary voltage: 3k V (ESI-); Ion source temperature 150℃; Desolvation temperature: 500℃; Desolventizing gas stream: 1000 L/Hr.

The raw data files generated by UPLC-MS/MS were processed using MassLynx software (Waters) and peak finding, integration, calibration, and quantification were performed for each metabolite. Mass spectrometry-based quantitative metabolomics referred to the comparison of an unknown substance with a set of standard samples of known concentration to determine the concentration of a substance in an unknown sample. For most analyses, a linear relationship between the instrument response and concentration was calculated to obtain y = ax + b (y represented the instrument response, where a was the slope/or sensitivity, b was the descriptive background constants). The concentration (x) of the unknown metabolite was calculated by this formula. Quantification of metabolites by LC/MS/MS were available in the Supplementary Table 7.

### Statistical Analysis

All statistical analysis and graphic illustrations were performed with Microsoft excel (office 365), or R (www.r-project.org/). Statistical significance was calculated with a t-test between the means of two groups. All quantification graphs were presented as mean ± standard deviation from at least three or more independent experiments. Statistical significance is defined as *p* < 0.05.

## Results

### PATZ1 is essential for pluripotency maintenance of hESCs

**Figure 1:**
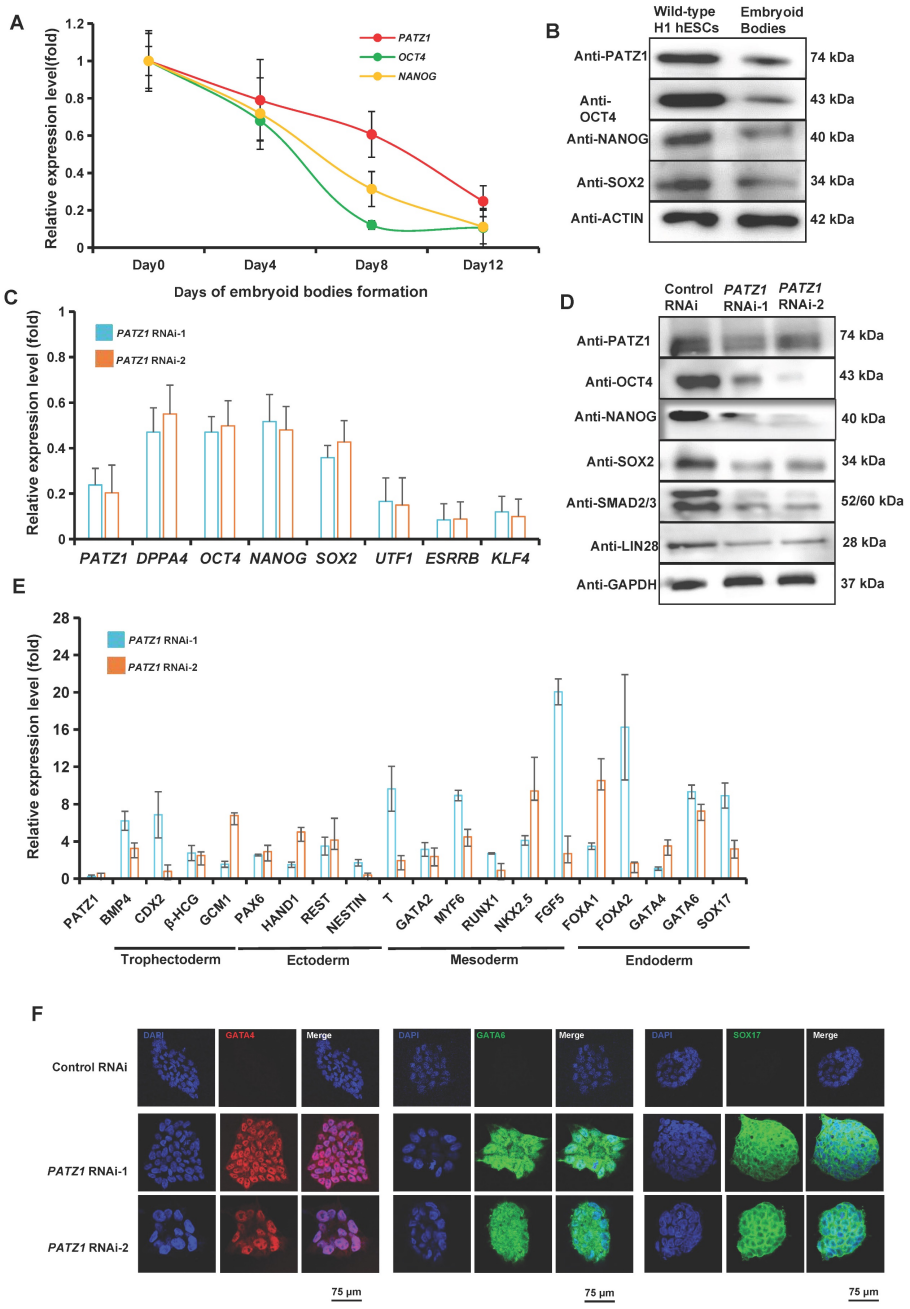
** PATZ1 is required for hESC pluripotency.** (A) mRNA level of *PATZ1* was downregulated in hESC cells upon differentiation. The level of the *PATZ1*, *OCT4* and *NANOG* were compared to undifferentiated wild-type H1 hESCs and normalized against *β-ACTIN*. *OCT4* and *NANOG* served as positive controls during EB formation (B) Protein level of PATZ1 was significantly decreased upon EB formation on Day 14. (C) mRNA levels of pluripotency genes, *DPPA4*, *OCT4*, *NANOG*, *SOX2*, *UTF1*, *ESSRB* and *KLF4* were dramatically decreased upon *PATZ1*-RNAi. hESCs transfected with empty vector were used as control. The expression of the genes in control cells was arbitrary considered “1”. The relative level of each mRNA was compared with Control-RNAi cells. (D) Depletion of PATZ1 caused reduced protein levels of pluripotency genes, OCT4, NANOG, SOX2, SMAD2/3 and LIN28. GAPDH served as loading control. (E) Deficiency of *PATZ1* activated lineage specific gene expression, particularly in mesoderm and endoderm. The expression of the genes in control cells was arbitrary considered “1”. The relative level of each mRNA was compared with Control-RNAi cells. (F) Immunostaining visualized that *PATZ1* RNAi caused representative developmental genes, GATA4 (Left), GATA6 (Middle) and SOX17 (Right) expression. Scale bar=75 μm. All data were presented as mean ± SD (n=3).

Immunostaining assay was performed to visualize the distribution of endogenous PATZ1 using an anti-PATZ1 antibody in hESCs. PATZ1 was mainly localized in the nucleus of wild-type H1 hESCs (Supplementary Fig. 1A). The protein fractions isolated from cytoplasm and nucleus confirmed that PATZ1 was dominantly distributed in the nucleus of the cell (Supplementary Fig. 1B). These observations indicate that as a zinc finger protein, PATZ1 is mainly located in the nucleus and may function as a transcription factor in hESCs. Next, to examine whether PATZ1 is associated with hESC pluripotency, we induced hESCs to differentiate by embryoid bodies (EBs) formation in a suspension culture system. Gene expression levels in EB cells was determined for each two days by RT-qPCR. As expected, mRNA expression of *PATZ1* was decreased by around 50% on Day 8 of the EB formation progress as *OCT4* and *NANOG* (Fig. 1A). Suspended EB cells were subsequently transferred onto a 0.1% gelatin-coated dish. *PATZ1* level was further dropped to 30% on Day 12 of EB formation. Consistently, the protein level of PATZ1 was significantly diminished on Day 14 of the EB formation progress (Fig. 1B). The downregulation of PATZ1 is considered as a rapid response of withdrawing OCT4, SOX2 and NANOG. Overall, our results demonstrated that PATZ1 is an essential pluripotency factor in hESCs.

PATZ1 depletion by *PATZ1* RNAi was used to determine the role of PATZ1 in hESC pluripotency. H1 hESCs were transfected with two independent sh*PATZ1* plasmids (*PATZ1* RNAi-1 and *PATZ1* RNAi-2). After transfecting with both plasmids, the mRNA level of *PATZ1* was significantly downregulated, as well as some other pluripotency genes, including *DPPA4*, *OCT4*, *NANOG*, *SOX2*, *UTF1*, *ESSRB,* and *KLF4* (Fig. 1C). The protein levels of representative master pluripotency regulators (OCT4, NANOG, SOX2, SMAD2/3 and LIN28) were reduced correspondingly (Fig. 1D). The downregulation of pluripotency genes caused by *PATZ1* knockdown revealed that depletion of PATZ1 may destroy OCT4/NANOG transcriptional circuity which governs pluripotency in hESCs 5, 45, 46. The AP staining additionally indicated that *PATZ1* knockdown resulted in morphology changes from typical ES morphology to fibroblast-like cell morphology with a clear reduction of alkaline phosphatase activity (Supplementary Fig. 1C). Concordantly, our results supported that PATZ1 plays a role in pluripotency regulation in hESCs.

Since suppression of pluripotency-associated genes causes the activation of developmental genes, we examined the mRNA level changes of typical germ layer (trophectoderm, ectoderm, mesoderm, and endoderm) lineage genes upon *PATZ1* knockdown (Fig. 1E). As expected, the RT-qPCR results showed depletion of *PATZ1* activated major lineage genes, including trophectoderm markers: *BMP4*, *CDX2*,* HCGβ*, and *GCM1* (2-6 folds); ectoderm markers: *PAX6*, *HAND*1 and *REST* (2-4 folds). There was a substantial upregulation of mesoderm markers: *MYF6* (8 folds), *NKK2.5* (10 folds) and *FGF5* (20 folds), and endoderm markers: *FOXA1* (10 folds), *FOXA2* (14 folds), *GATA6* (4 folds), and *SOX17* (8 folds). The significant upregulation of mesoderm and endoderm markers suggested that *PATZ1* contributes into suppressing these lineage genes to maintain hESC pluripotency. To look for differentiation bias, we induced the wild-type H1 hESCs and *PATZ1*-KD stable cells into EB cells by spontaneous differentiation (Supplementary Fig. 1D). Our subsequent RT-qPCR results confirmed that as compared to the EBs induced from wild-type hESCs, EBs derived from *PATZ1*-depleted hESCs preferred to differentiate into mesoderm and endoderm (Supplementary Fig. 1E). To evaluate protein level of lineage markers upon *PATZ1* knockdown, we performed immunostaining in both negative control- and *PATZ1*-knockdown cells. The protein levels of representative lineage markers, GATA4 (Fig. 1F Left), GATA6 (Fig. 1F Middle) and SOX17 (Fig. 1F Right) was visualized by immunostaining stained with specific antibodies. PATZ1-depleted cells clearly displayed strong fluorescence while very low signals could be detected in the control cells. These results affirmed that loss of PATZ1 induces hESC differentiation.

To extensively investigate the global gene expression changes upon *PATZ1* knockdown in hESCs, we isolated RNA from control and *PATZ1* knockdown cells (three replicates each) and performed RNA-seq. The RNA-seq data was analysed by a publicly accessible platform - the CSI NGS Portal (https://csibioinfo.nus.edu.sg/csingsportal). To validate the reliability of RNA-seq results, we randomly selected nine genes and performed RT-qPCR (Supplementary Fig. 2A). Overall, the RNA-seq results mutually validated the experimental data (Fig. 1). Of note, from RNA-seq data, many well-defined pluripotency genes were downregulated (Supplementary Fig. 2B), while developmental genes were upregulated (Supplementary Fig. 2C). Taken together, our data demonstrated that PATZ1 plays an essential role in pluripotency maintenance in hESCs and depletion of PATZ1 drives hESCs into differentiation.

### Genome-wide location analysis of PATZ1 shows that PATZ1 is integrated into the OCT4/NANOG transcriptional network

**Figure 2:**
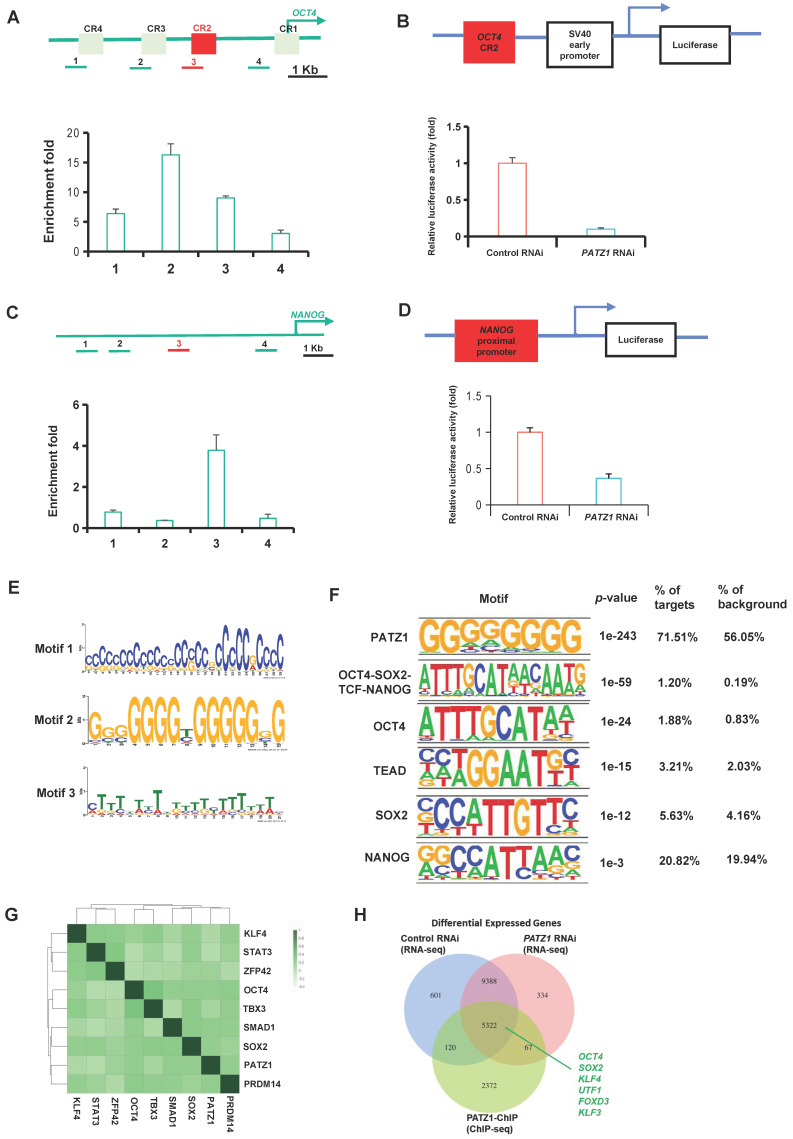
** Genome-wide binding sites of PATZ1.** (A/C) Top: Location of specific primers were mapped to promoter region of *OCT4*/*NANOG* loci. Bottom: PATZ1 binds to the distal enhancer of *OCT4*/ *NANOG* loci. (B) Top: CR2 region of *OCT4* loci was cloned into the downstream of luciferase gene driven by SV40 promoter. Bottom: Luciferase activity of CR2 in *PATZ1* RNAi-1 treated cells. (D) Top: The *NANOG* proximal promoter was cloned into the luciferase construct. Bottom: Luciferase activity of *NANOG* proximal promoter in *PATZ1* RNAi-1 treated cells. (E) Computed putative binding top three enriched motifs in PATZ1 binding sites. (F) PATZ1 binding motifs were highly enriched in binding regions of other pluripotency factors, OCT4, TEAD, SOX2 and NANOG. (G) Co-occurrence frequency of transcription factors at multiple binding loci. Degree of correlation was indicated as the shades of color. (H) Overlapping of PATZ1 in ChIP-seq putative binding targets, and the differential expressed genes from RNA-seq dataset. Pluripotency genes, *OCT4*, *SOX2*, *KLF4*, *UTF1*, *FOXD3*, and *KLF3*, were found in both ChIP-seq and RNA-seq dataset. All data were presented as mean ± SD (n=3).

We hypothesized that PATZ1 regulates transcriptions of *OCT4* and *NANOG* since their expressions were significantly reduced upon *PATZ1* RNAi. The binding sites of PATZ1 at *OCT4* and *NANOG* genomic loci were determined by ChIP. PATZ1 ChIP DNA with gene-specific primers were analyzed along with the* OCT4* (Fig. 2A Top) and *NANOG* (Fig. 2C Top) loci using real-time PCR. There were significant enrichment folds in conserved regions (CR) of distal promoter sites of *OCT4* in which known as proximal enhancers (Fig. 2A Bottom). According to our ChIP-qPCR result, PATZ1 strongly enriches the distal promoter of OCT4. CR2, CR3 and CR4 regions were then cloned into a luciferase reporter plasmid to test whether PATZ1 can regulate transcription of *OCT4.* As expected, *PATZ1*-depleted cells showed a significant reduction of luciferase activity as compared to the control cells in CR2 region (Fig. 2B) and in CR4, CR3 regions (Supplementary Fig. 3A and 3B). Similar results were observed on the *NANOG* proximal promoter (Fig. 2C and 2D). Together, PATZ1 is essential for hESC pluripotency via directly modulating the transcription of pluripotency genes *OCT4/NANOG*.

PATZ1 ChIP-seq was used to determine genome-wide locations of PATZ1 in hESCs. Genomic regions defined by multiple overlapping DNA fragments enriched by PATZ1 ChIP were considered as putative binding sites. To validate the reliability of these putative binding sites, we first putative binding regions with qPCR based on the peak height of 8-, 9-, 10, 11-folds from ChIP-seq dataset. A threshold value of 2 enrichment folds in qPCR was considered as a real binding site (Supplementary Fig. 4A). Genomic sequence reads were mapped to the hg38 human genome assembly. To avoid the non-specific background from high-throughput sequencing, a peak height has 5 folds or above was defined as biologically authentic binding site. By this criterion, a total of 4719 PATZ1 binding sites with high confidence were identified. Considering the immunostainings that PATZ1 was mainly localized in the nucleus, it was not surprising that PATZ1 occupied abundant DNA regions across the human genome. The majority of these putative binding sites were located in promoters (< 3 kb from TSS) and intronic regions (Supplementary Fig. 4B Left). ngsPlot supplementally indicated the intensive enrichment location of PATZ1 on the TSS of promoter regions (Supplementary Fig. 4B Right). These suggested that PATZ1, as a transcription factor, favors promoter regions of its downstream genes to modulate transcription activation or repression.

ChIP-seq data through bioinformatic computation was performed to search for the binding motif of PATZ1 in hESCs. On average, approximately 500 bp of PATZ1 binding peaks distributed in a 50-percentile position of summits surrounding the PATZ1 motif (Supplementary Fig. 4C). Top three significant enriched motifs were selected in accordance with E scores (Fig. 2E). Motif 1 consisted of cytosine-rich (C-rich) 29 nucleotides. Similarly, Motif 2 harboured large region of guanine-rich (G-rich), while Motif 3 was highly enriched with thymine (T-rich). We accordingly figured out the consolidated PATZ1 motif which was enriched with GC fragments. The conserved motif in human ESCs were consistent with previous studies by our laboratory 22, 27. The high affinity to cytosine and guanine shall be attributed to the two AT-hook domains in the central region, and a C2H2-ZF domain at the C-terminal in PATZ1^19^.

To explore whether PATZ1 may interact with other pluripotency regulators in hESCs, we examined the binding sites of other transcription factors where PATZ1 enriched. As expected, PATZ1 binding regions indeed were highly enriched with multiple master pluripotency regulators (*OCT4, TCF, TEAD, SOX2* and* NANOG*) (Fig. 2F). These PATZ1 binding motifs were highly enriched in these regions along with more moderate enrichment for representative pluripotency factors. To determine whether PATZ1 is part of the core pluripotency circuit of hESCs, PATZ1 was clustered with other pluripotency factors based on their genomic binding sites, suggesting that PATZ1 indeed co-occupies many genomic sites with other transcription factors (Fig. 2G). The strong correlation of each transcription factor pair indicated that PATZ1 is integrated in the whole pluripotency regulatory network in hESCs. Master pluripotency regulator genes (*OCT4*, *SOX2*, *KLF4*, *UTF1*, *FOXD3* and *KLF3*) co-existed in both ChIP-seq and RNA-seq dataset (Fig. 2H). The cross-comparison of RNA-seq and ChIP-seq refines that PATZ1 is an essential component in the whole transcription network of hESCs. Endogenous co-IP additionally showed the physical interaction of PATZ1/OCT4/SOX2/NANOG (Supplementary Fig. 4D). In fact, except for the DNA-binding, protein-protein interaction of pluripotency factors indispensably contributes to complement the ESC pluripotency which provides feedback signaling through the protein network 47. Taken together, we suggest that PATZ1 is a key node in OCT4/SOX2/NANOG transcriptional network.

We further annotate the putative downstream genes of PATZ1 gene targets based on our PATZ1 ChIP-seq data, Gene Ontology (GO) analysis showed that many putative gene targets enriched in GO term were related to differentiation, such as nervous system development and anatomical structure development (Supplementary Fig. 4E). Interestingly, PATZ1 may provide RNA-editing sites for other pluripotency regulators to adapt the transcriptional cues since *DPPA4* was over-edited while *LIN2A* and *NANOG* were under-edited in the *PATZ1*-knockdown cells (Supplementary Fig. 4F). Together, PATZ1 is an indispensable pluripotency factor in the core transcription circuity of hESCs and functionally acts as a transcriptional suppressor to inhibit developmental genes to intensify human ESC pluripotency.

### Depletion of PATZ1 results in ES cell proliferation defects and apoptotic cell death

**Figure 3:**
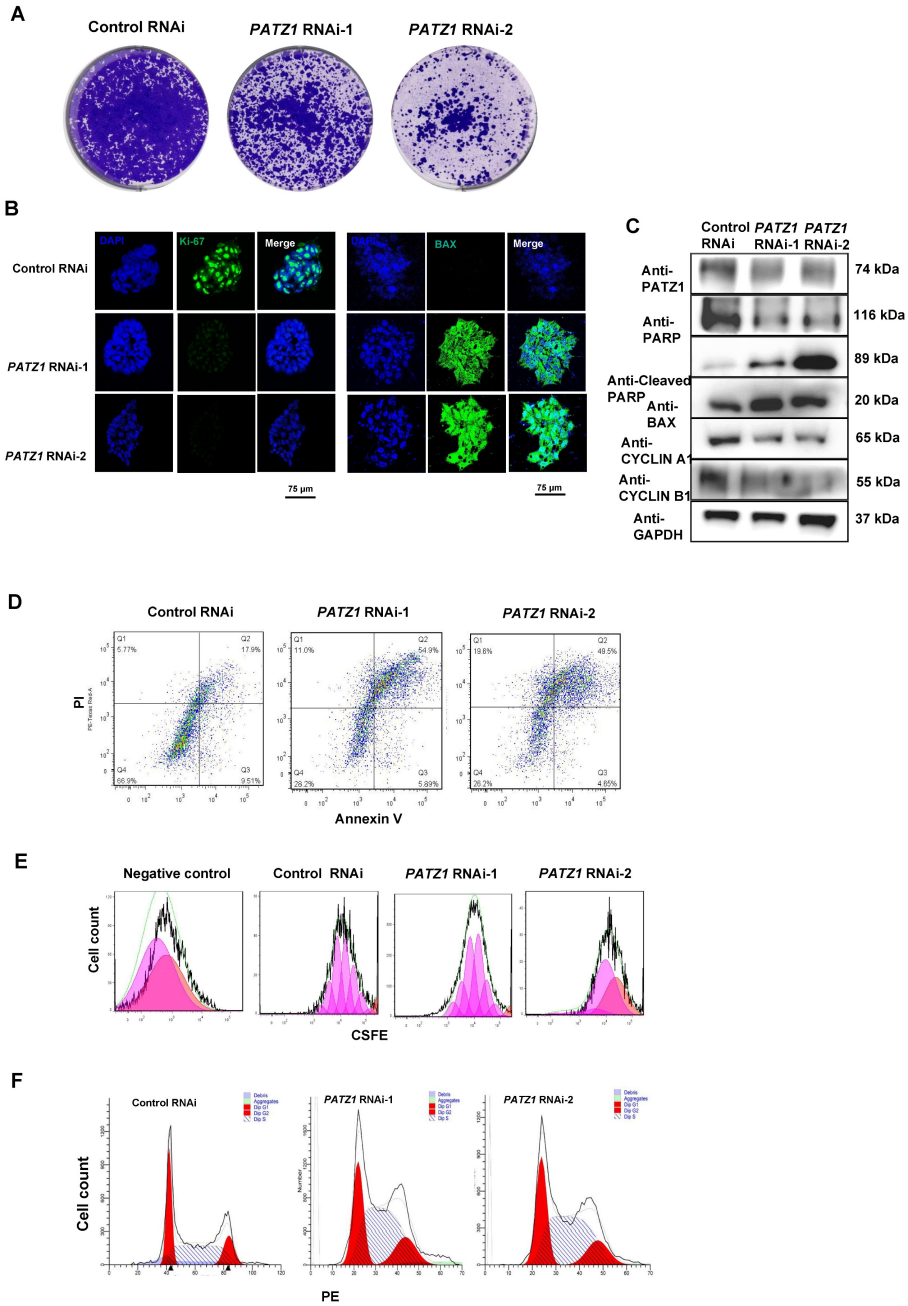
** PATZ1 is necessary for hESC proliferation.** (A) Stable *PATZ1*-KD hindered the cell colony formation ability of hESCs. (B) Immunostaining visualized that depletion of PATZ1 decreased the cell proliferation marker, Ki-67 (Left) expression, and promoted pro-apoptosis marker, BAX (Right) expression. (C) Knockdown of PATZ1 reduced protein levels of anti-apoptosis marker RARP, and cell cycle makers, CYCLIN A1 and CYCLIN B1, while facilitated the pro-apoptosis markers, Cleaved RARP and BAX. GAPDH served as loading control. (D) A representative apoptosis of control- and *PATZ1*-RNAi cells were determined by flow cytometry with Annexin-V and PI staining. (E) A representative cell division of control- and *PATZ1*-RNAi cells were measured by flow cytometry with CSFE staining. Proportions of cell population were labeled as cell division. (F) A representative cell cycle analysis of control- and *PATZ1*-RNAi cells were measured by flow cytometry with PE staining. Red color represented the G0/G1 phase, and yellow color illustrated the G2/M phase. The intermediate area of two peaks depicted the S-phase window.

Notably, depletion of PATZ1 rapidly inhibited the cell viability. Our AP staining (Supplementary Fig. 1C) and MTT assays (Supplementary Fig. 5A) indicated that the cell viability was strikingly reduced upon *PATZ1* RNAi. Colony formation assay additionally demonstrated that chronic *PATZ1* knockdown decelerated the cell proliferation as compared to control cells (Fig. 3A). Besides, our Wright-Giemsa staining result indicated that apoptotic cells were obviously shrunk in which the chromatin was agglutinated and marginalized. (Supplementary Fig. 5B). Importantly, cell proliferation marker Ki-67 was down-regulated, while cell apoptosis marker BAX expression was increased upon *PATZ1* knockdown (Fig. 3B). CYCLIN A1, CYCLIN B1 and PARP were dramatically decreased whereas pro-apoptosis markers (BAX, and cleaved PARP) were significantly upregulated upon *PATZ1* knockdown (Fig. 3C). Collectively, we concluded that depletion of PATZ1 impaired cell proliferation and induced apoptotic cell death.

A set of flow cytometry assays was employed to interpret above phenotypic results, including apoptosis, CSFE and cell cycle assays. The number of dead and apoptotic cells, as sorted by Annexin V, were significantly increased in *PATZ1* depleted cells (Fig. 3D and Supplementary Fig. 5D), suggesting that depletion of PATZ1 triggered apoptosis. The CSFE incorporation assay was used to determine the proliferative status and cell cycle duration of hESCs 37. As expected, the cell proliferative rate of hESCs was compromised and the cell cycle was arrested (Fig. 3E and Supplementary Fig. 5E). Consistently, PATZ1 depleted cells showed a shorter S-phase window, indicating the amount of duplicating DNA was decreased during cell division (Fig. 3F and Supplementary Fig. 5F). RNA-seq results consistently showed that PATZ1 was significantly involved in various cell cycle-regulatory phases, including cell cycle checkpoints and mitotic G1 phase and G1/S transition (Supplementary Fig. 5G). Together, PATZ1 is critical to preserve a normal cell cycle and prevent apoptosis in hESCs.

### Depletion of PATZ1 disrupts mitochondrial functions

**Figure 4:**
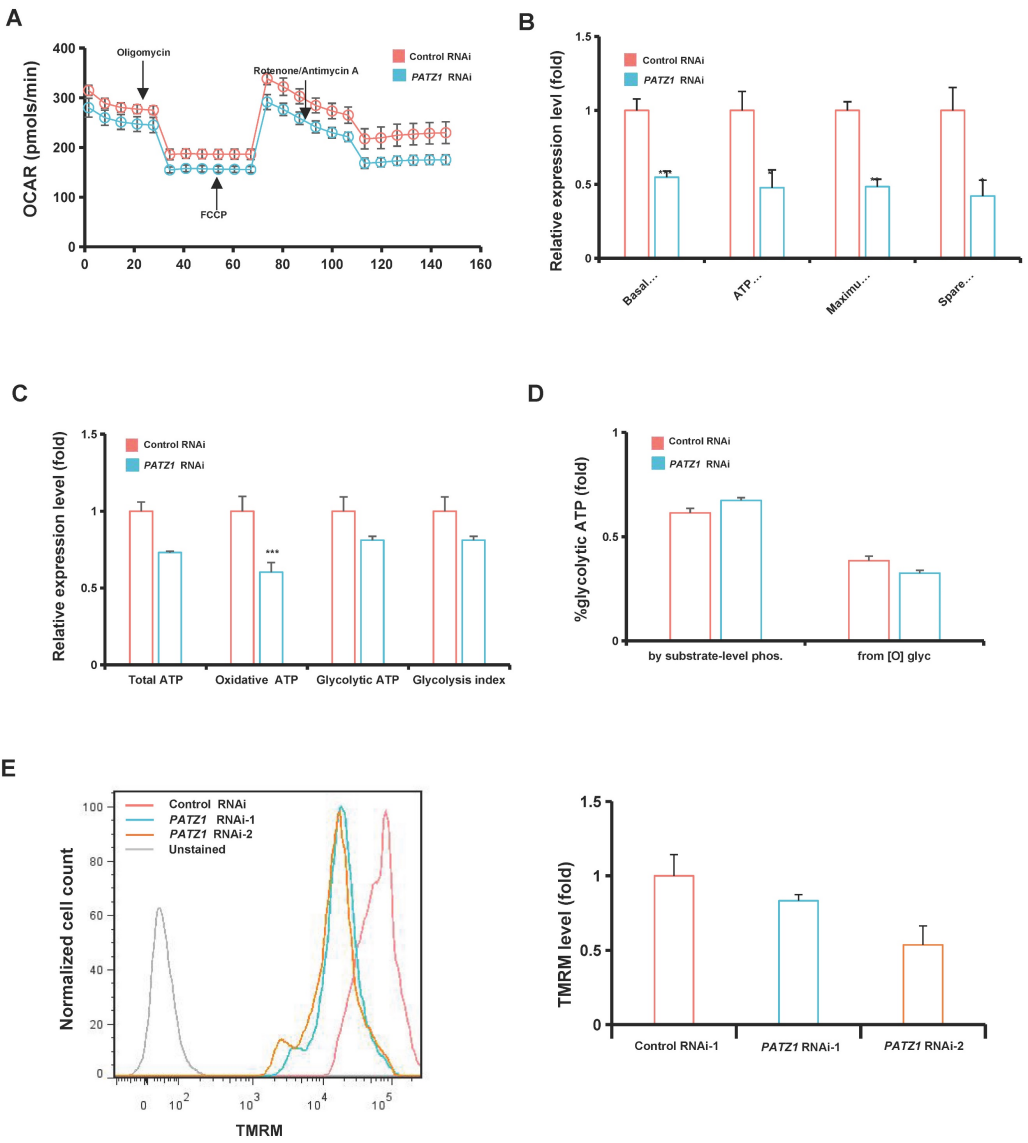
** Depletion of PATZ1 blocks the bioenergetic transition from TCA cycle.** (A) A representative graph for Seahorse measurements of OCR. (B) Quantification of OCR parameters. (C) Theoretical ATP production from oxidative phosphorylation glycolysis. (D) Relative contribution of glycolysis to total ATP production. (E) Left: Mitochondria membrane potential of control- and *PATZ1*-RNAi cells were measured by flow cytometry with TMRM staining assay. Right: Summary of each mitochondrial membrane potential labeled with the fluorescence intensity of TMRM. For Figure 4A-4D, *PATZ1* RNAi-1 was used. For figure 4E-4F, both *PATZ1* RNAi-1 and *PATZ1* RNAi-2 were used. All data were presented as mean ± SD (n=3). Significance: * *p* <≤ 0.05, **p ≤ 0.01, *** *p* ≤ 0.001.

Having demonstrated that PATZ1 is required for hESC survival, we speculated that depletion of PATZ1 probably impairs the metabolic program in hESCs. We first quantified the rate of ATP production from glycolytic and mitochondrial system simultaneously by measuring oxygen consumption rate (OCR) and extra cellular acidification rate (ECAR) using an Agilent Seahorse XF Analyzer. OCR represents the rate of mitochondrial oxidative phosphorylation, while extracellular acidification rate (ECAR) indicates the glycolysis efficiency in the cells. Seahorse results indicated that *PATZ1* knockdown largely altered OXPHOS (Fig. 4A and 4B). The downregulated OCAR indicated a mitochondrial suppression upon depletion of PATZ1. In contrast to OCAR, the decline of ECAR parameters was not significant (Supplementary Fig. 6A and 6B), suggesting that the significant reduction of OCAR was dominantly caused by OXPHOS suppression. In turn, ESCs lacking PATZ1 relied on exploiting glycolysis when mitochondrial function was damaged. Furthermore, oxidative ATP production was approximately decreased by 40% while the glycolysis index was dropped by 20% in PATZ1-depleted cells (Fig. 4C). So oxidative ATP contributed to a proportional reduction in the total ATP of the cells. Moreover, since substrate-level phosphorylation in the cytoplasm behaved as the major ATP production resource when calculating the glycolytic ATP production, mitochondrial oxidation of NADH transported from the cytoplasm was gradually decreased upon *PATZ1* knockdown (Fig. 4D). Unexpectedly, the transcriptomic and protein changes of mitochondrial biogenesis genes (Supplementary Fig. 6C and 6D) were upregulated. This was probably attributed to the relatively stable load in mitochondria so that they did not exhibit consistent changes 44. Collectively, absence of PATZ1 impaired the mitochondrial respiration. hESCs were forced to exploit glycolysis as the energy resource in responded to mitochondrial dysfunction.

Next, we investigated whether PATZ1 directly regulated the mitochondrial function. The mitochondrial membrane potential is quantified by flow cytometry using tetramethylrhodamine, methyl ester (TMRM). As expected, the TMRM level were significantly downregulated in PATZ1 depleted cells (Fig. 4E). In addition, by labeling JC-1, *PATZ1* knockdown cells exhibited intensive JC-green fluorescence while the control cells were mainly stained with JC-red fluorescence (Supplementary Fig. 6E), indicating a reduction of mitochondrial membrane potential upon PATZ1 depletion. MT-DNA copy numbers were significantly reduced in *PATZ1* knockdown cells (Supplementary Fig. 6F). A comparison of PATZ1 proteins from different mammal species indicated a redox-sensitive cysteine which was probably caused by PATZ1 (Supplementary Fig. 6G). Taken together, we suggest that PATZ1 is required to sustain regular mitochondrial function.

### Multi-omics analysis reveals the involvement of PATZ1 in maintenance of cellular homeostasis

**Figure 5:**
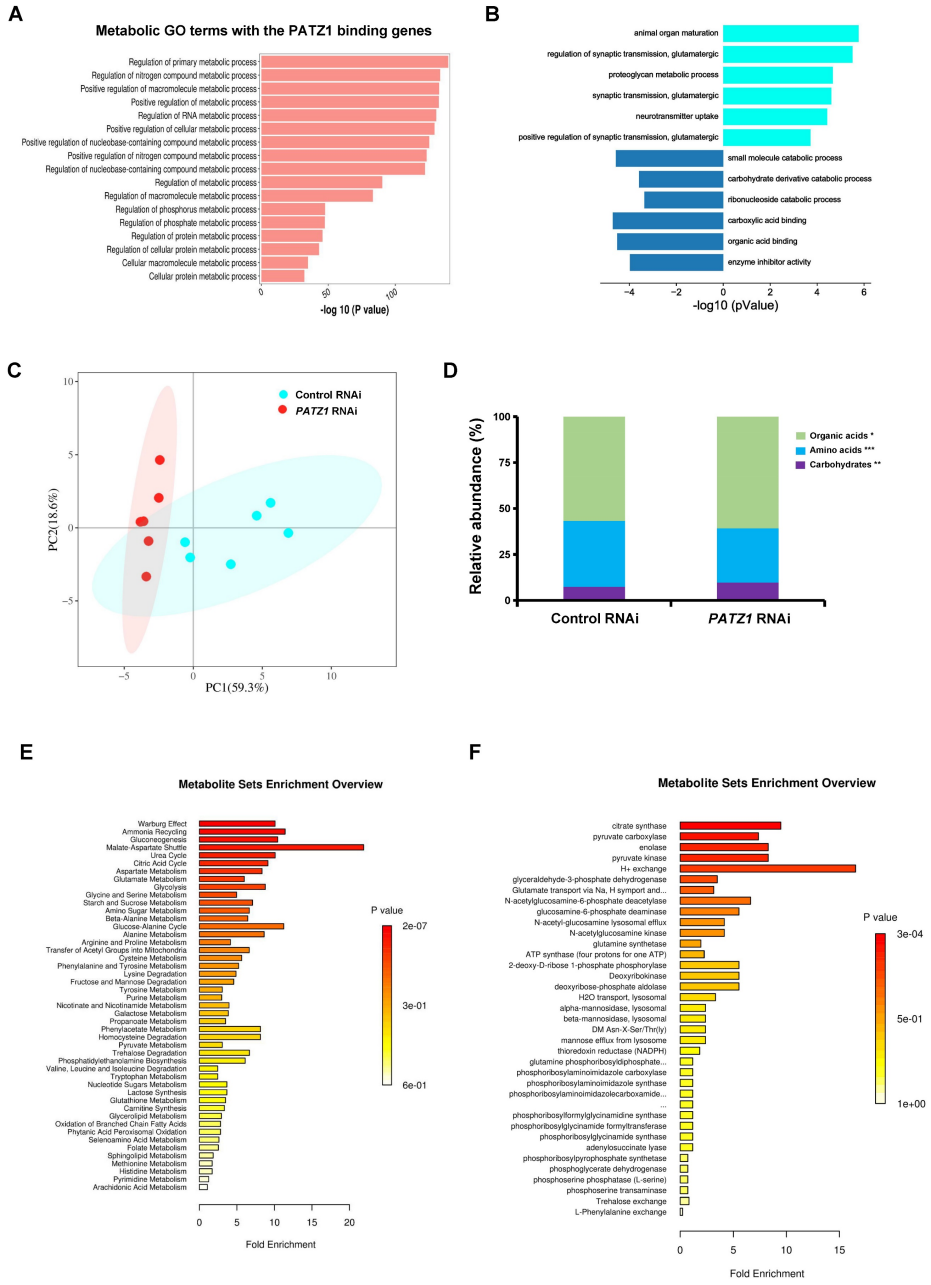
** Multi-omics analysis reveals that PATZ1 regulates ES cell homeostasis.** (A) Metabolic GO term with PATZ1 binding sites from the ChIP-seq data. (B) Enrichment pathway of metabolic GO terms corresponding to up-regulated gene and down-regulated genes in *PATZ1* knockdown hESCs compared with control cells. (C) PCA of the targeted metabolome of control- and *PATZ1-* RNAi group. (D) Relative abundance of detectable metabolite classes in control- and *PATZ1-* RNAi groups were shown in stacked bar chart. (E) Metabolite set enrichment analysis of differentially abundant metabolites using pathway-associated metabolite sets (Pathway-associated metabolite sets). Horizontal bars represented pathway fold enrichment and the color gradient indicate statistical significance. (F) Metabolite set enrichment analysis of differentially abundant metabolites using pathway-associated metabolite sets (Predicted metabolite sets). Horizontal bars represented pathway fold enrichment and the color gradient indicate statistical significance. All data were presented as mean ± SD (n=6).

Having determined that PATZ1 is involved in ATP production, we tested whether PATZ1 is required to maintain a steady metabolic homeostasis. Gene expression changes of a set of metabolism-regulatory genes upon *PATZ1* knockdown were examined. Indeed, depletion of PATZ1 caused a global reduction of genes which were associated with diverse metabolic processes, including glycolysis, fatty acids oxidation, TCA cycle, lipid metabolism, and amino acid biosynthesis (Supplementary Fig. 7A). Consistently, proteins encoded by these genes including GLUT1, PHD, G6PD, LDJA, HK1, HK2 and PKM2 were significantly downregulated (Supplementary Fig. 7B). ChIP-seq results indicated that PATZ1 bound to genomic regions of most representative metabolic genes, such as *PFKP* (fold enrichment = 10.3522), *PHGDH* (fold enrichment = 5.02481), *ELOVL5* (fold enrichment = 4.49494), *PTGR1* (fold enrichment = 4.52675), *PKM* (fold enrichment = 4.51325) and *GLUD1* (fold enrichment = 4.0351) (Supplementary Fig. 7C). GO analysis indicated that many putative downstream genes of PATZ1 were enriched for metabolic processes, such as “Regulation of primary metabolic process” (Enrichment = 1.0357E-60, Target genes in term = 2469), and “Regulation of nitrogen compound metabolic process” (Enrichment = 1.554E-58, Target genes in term = 2398) (Fig. 5A). GO analysis of the RNA-seq dataset indicated that genes related to neurodevelopmental and glutamatergic processes were upregulated, whereas genes involved in some metabolic processes were downregulated in PATZ1-depleted hESCs (Fig. 5B). Overall, we conclude that PATZ1 depletion not only results in cell fate change but also causes metabolic shifts.

A targeted quantitative metabolomics analysis was measured by LC/MS/MS in tern of profiling the metabolic process. As indicated, a principal-component analysis (PCA) of full dataset indicated an intensive correlation structure in the metabolomic data across both group with the first principal component (PC1) largely capturing the effect of *PATZ1*-RNAi with the first two PCs explaining 77.9% of the variation (Fig. 5C). The relative abundance of metabolites was significantly changed upon *PATZ1* knockdown. Notably, the abundance of amino acids in PATZ1-depleted cells were relatively increased compared to control (Fig. 5D and Supplementary Fig. 7D). So, ES cells might be driven to rely more on glucose metabolism upon mitochondrial dysfunction. The metabolite set enrichment analysis using pathway-associated metabolite sets (SMPDB) identified 47 enriched metabolic pathways, and 18 of them were significant. The three most significantly enriched KEGG pathways included “Warburg effect” (*p*=1.51E-07, Hits=8), “Ammonia Recycling” (*p*=3.55E-05, Hits=5), and “Gluconeogenesis” (*p*=5.59E-05, Hits=8). The abundance of glucose was significantly enriched in KEGG pathways, including “Gluconeogenesis” (*p*=5.59E-05, Hits=5), and “Glycolysis” *(p*=3.92E-03, Hits=3) (Fig. 5E). Besides, our pathway enrichment analysis using Pathway-associated metabolite sets (Predicted metabolite sets) was shown in Fig. 5F. Consistent with the role of PATZ1 in cell fate decision, “Citric Acid Cycle” (*p*=6.33E-04, Hits=4) and “Transfer of Acetyl Groups into Mitochondria” (*p*=3.43E-02, Hits=2) were involved in the significant KEGG terms. Collectively, our metabolomics data demonstrated a multi-functional role of PATZ1 in hESC metabolism.

### PATZ1 balances glycolysis and OXPHOS in hESCs

**Figure 6:**
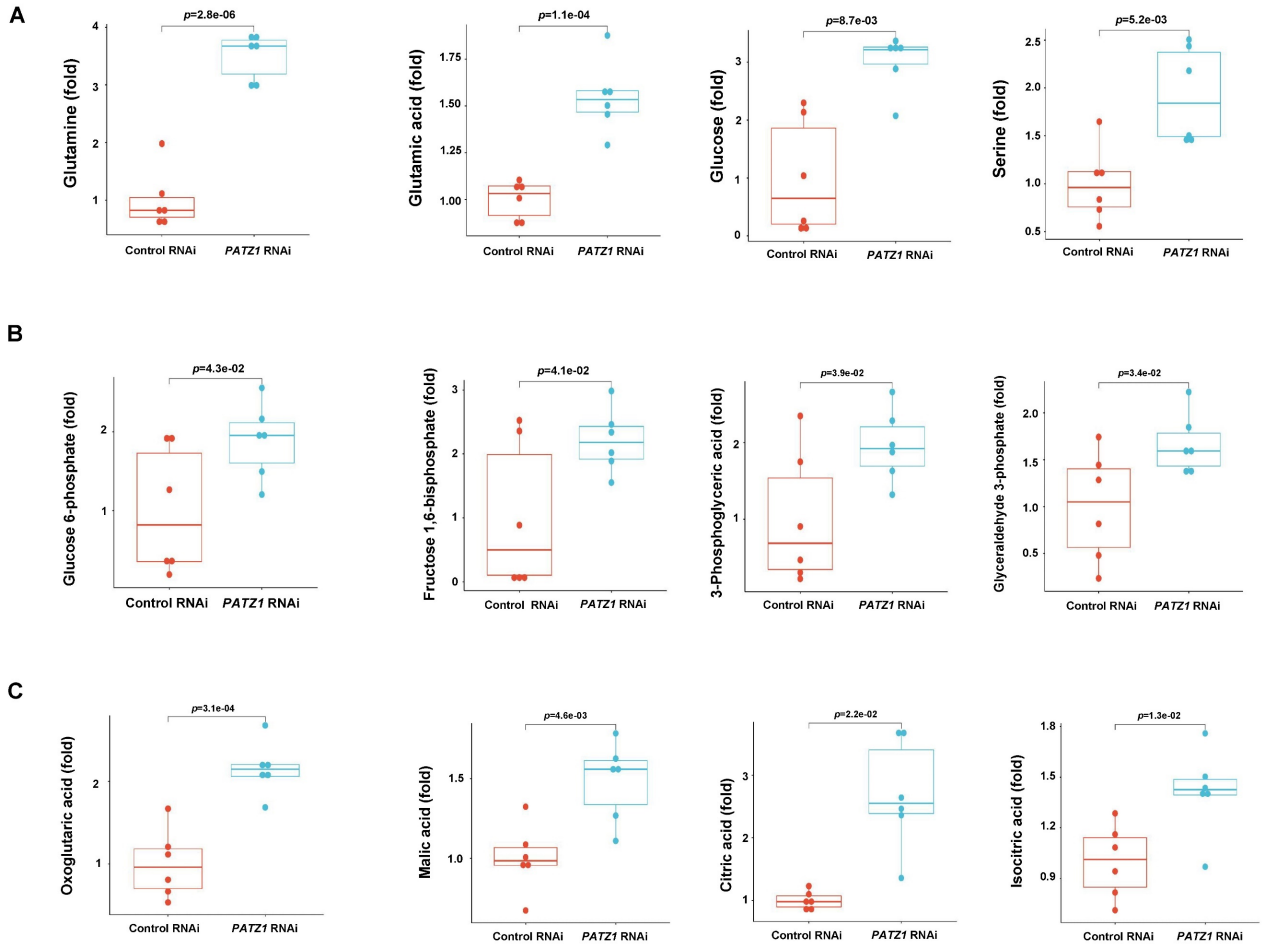
** Quantification of differential metabolites by LC/MS/MS.** (A) Boxplot of main metabolite broad classes. (B) Boxplot of significantly changed glycolysis metabolites. (C) Boxplot of significantly changed TCA cycle metabolites. PATZ1 RNAi-1 was used to figure 6A-6C. All data were presented as mean ± SD (n=6).

A total of 26 glycolytic metabolites were detectable by LC/MS/MS and 17 of them were significantly upregulated in the *PATZ1*-RNAi cells. Among all detectable intermediates, major metabolic end-products including glutamine (*p*=2.8E-06), glutamic acid (*p*=1.1E-04), glucose (*p*=8.7E-3) and serine (*p*=5.2E-3) were significant upregulated in the* PATZ1*-knockdown cells (Fig. 6A), suggesting that PATZ1 affected multiple metabolic pathways. Next, we sort to identify the metabolic pathways ESCs rely on when PATZ1 was depleted. Most glycolytic metabolites were increased in the *PATZ1*-KD cells (Fig. 6B). This result may verify the qPCR results that depletion of PATZ1 suppresses the activity of most metabolic-regulatory genes (Supplementary Fig. 7A). The accumulation of glycolytic products in *PATZ1*-KD cells indicated that ESCs were forced to consume glucoses for energy production. Lactate production was measured because cell differentiation is associated with conversion of glucose to lactate under aerobic condition, displaying a phenomenon known as aerobic glycolysis 48. Lactate synthesis in the *PATZ1*-KD cells was relatively higher as compared to control, indicating a resultant of aerobic glycolysis in the *PATZ1*-KD cells, rather than OXPHOS alone (Supplementary Fig. 7D). Moreover, the TCA cycle intermediates were upregulated in the *PATZ1*-KD cells. This was consistent with that ESC differentiation was associated with the upregulation of TCA cycle metabolites (Fig. 6C) 49. Upon differentiation, ESCs oxidize most of glycolysis-derived pyruvate via OXPHOS, and the upregulated TCA cycle metabolites are resultant of OXPHOS in mitochondria 50. Overall, we concluded that depletion of PATZ1 activates aerobic glycolysis to support the ESC differentiation.

**Figure 7:**
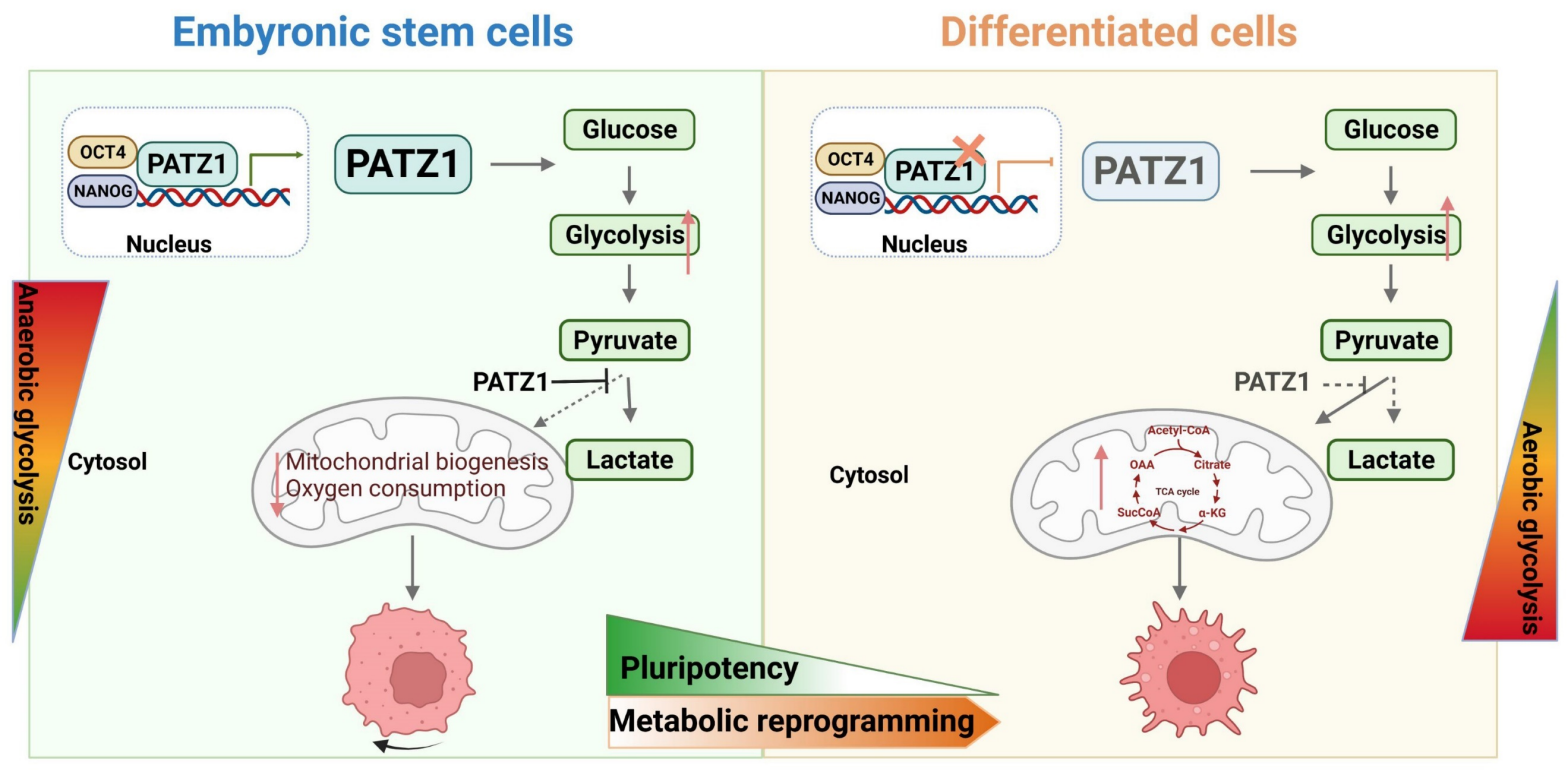
** Schematic illustration of PATZ1 in undifferentiated and differentiated hESCs.** Left: In undifferentiated ESCs, PATZ1 maintains the pluripotent state through interacting with OCT4 and NANOG. Undifferentiated ESCs mainly use glycolysis for ATP production, and PATZ1 suppresses the mitochondrial biogenesis. Right: Loss of PATZ1 initiates ESC differentiation with TCA cycle upgradation. Insufficient PATZ1 destroys the balance of glycolysis and OXPHOS. Absence of PATZ1 forces ESCs heavily rely on glycolysis that compensates the energy deficiency caused by mitochondrial damage during differentiation.

In summary, we establish a fundamental role of PATZ1 in hESCs (Fig. 7). In undifferentiated ESCs, PATZ1 binds to the promoter regions of *OCT4* and *NANOG*. It is an essential member of transcriptional network to contribute into self-renewal and pluripotency. Meanwhile, PATZ1 suppresses the mitochondrial biogenesis and oxygen consumption in hESCs. Absence of PATZ1 triggers ESC differentiation which is associated with upgraded TCA cycle and OXPHOS. However, as depletion of PATZ1 results in mitochondrial dysfunction, ESCs are hence forced to rely on aerobic glycolysis to compensates the energy deficiency caused by mitochondrial damage. Therefore, our study provides novel insights into the important roles of PATZ1 in regulating homeostasis of hESCs.

## Discussion

In this study, we showed that PATZ1 is not only an essential pluripotency regulator of ES cell identity but also a mediator of ES cell homeostasis. Previous studies by our laboratory have highlighted that loss of Patz1 accelerates early lineage differentiation in murine ESCs 22. Our current study further confirms an essential role of PATZ1 in transcriptional regulation of stem cell pluripotency genes, since (a) knockdown of *PATZ1* suppresses a set of typical stem cell regulators; (b) upregulation of mesoderm and endoderm genes reveals that PATZ1 mainly suppresses these two germ layers to promote pluripotency; (c) *PATZ1*-knockdown hESCs exhibit cell differentiation morphology. Therefore, the downregulation of PATZ1 is considered as a cue for differentiation of hESCs. (d) PATZ1 positively regulates the transcriptions of *OCT4* and *NANOG* through binding to the upstream of their promoters and activates their transcription directly. The large region of GC and AT fragments in PATZ1 binding motif are evolutionally conserved across mouse to human. Additional strong protein-protein interactions among PATZ1, OCT4 and NANOG contribute to pluripotency maintenance.

hESCs exhibit a rapid cell cycle due to a selective reduction of the G1 phase 37. *PATZ1*-KD hESCs displayed a clear suppression of cell proliferation and loss of typical ESC features. This necessity of PATZ1 in ESC survival is like the regulatory role of PATZ1 in several cancer studies. PATZ1 depletion alters the cell cycle due to the shortened S-phase window. Our results are consistent with previous literature about the importance of PATZ1 in cell proliferation and embryonic development 19, 29. Apart from cell cycles, PATZ1 may have the potential redox activity. Cysteines presented in PATZ1 were evolutionarily conserved across species. Several cysteines are very adjacent to arginine and lysine. Given that cysteines are sensitive to oxidative modification, the redox activity of cysteines in PATZ1 is probably increased by positively vicinal charged amino acids 51. Overall, we provide evidence that depletion of PATZ1 affects cell growth in hESCs.

An important finding in this study is that loss of PATZ1 significantly causes mitochondrial dysfunction. We consider the upregulation of TCA cycle intermediates was a resultant of multiple metabolic resources. (1) The differentiation of ESCs caused by *PATZ1-*KD results in a concomitant increase in TCA cycle. (2) accumulated glycolytic metabolites promote acetyl CoA, and subsequently activate the citric acid and isocitric acid. (3) High levels of glutamine and glutamic acid supported the TCA cycle through αKG supply. Together with active glycolysis, loss of PATZ1 may induce aerobic glycolysis in ESCs. Compared to anerobic glycolysis, aerobic glycolysis is a more specific mode to describe glycolytic metabolism with recruitment of lactic acid even under aerobic conditions 49. If this is the case, cells may have to alternatively use other metabolic pathways to support cellular function. *PATZ*1-KD cells displayed a significant accumulation of glycolysis intermediates as compared to control cells, suggesting that glycolysis was still active even after PATZ1 was depleted. Similar observation was reported in another study 52. The cells may become more glycolytic-dependent than their normal counterparts. Therefore, there is an interesting dichotomy that mitochondrial function supports the ESC proliferation while glycolysis is required for pluripotency 11. Notably, while primed pluripotent stem cells prefer to maintain a low mitochondrial respiration state, naïve ESCs rely on both glycolysis and mitochondrial oxidation to maintain their pluripotent state. Therefore, we suggest that PATZ1 depletion disrupts mitochondrial function but compensates with increased glycolysis.

## Conclusion

In conclusion, PATZ1 regulates the pluripotent state of hESCs through binding to the distal enhancer of *OCT4* and proximal promoter of* NANOG* and regulating the transcription of *Oct4* and *Nanog*. PATZ1 maintains hESC pluripotency while suppressing mesodermal and endodermal genes. In addition, PATZ1 supports hESC proliferation through controlling the G1/S transition and inhibiting apoptosis. Importantly, PATZ1 balances glycolysis and oxidative phosphorylation to regulate hESC homeostasis. Taken together, PATZ1 plays multiple essential roles in hESCs.

## Supplementary Material

Supplementary figures and tables.
