# Supplementary material for: POZ/BTB and AT hook containing zinc finger 1 (PATZ1) suppresses differentiation and regulates metabolism in human embryonic stem cells
Source: Int J Biol Sci. 2024 Jan 21;20(4):1142–59. doi: 10.7150/ijbs.83927 (PMC10878140; doi:10.7150/ijbs.83927)
Supplement: Supplementary file 1 — Supplementary figures and tables. [file ijbsv20p1142s1.pdf]

Supplementary figure legends

Figure S1. PATZ1 is essential for hESC pluripotency. (A-B) Immunostaining of endogenous PATZ1 indicated the nucleus distribution of wild-type H1 hESCs (A) and confirmed by western blotting assay (B). DAPI served as nuclear staining. Scale bar = 100 μm. (C) A representative alkaline phosphatase staining of hESCs transfected with scrambled- and *PATZ1*- RNAi. (D) Inducing wild-type H1 hESCs (Left) and *PATZ1*-KD stable cells (Right) into EB cells. Scale bar = 100 μm. (E) EBs induced from PATZ1-depleted hESCs more likely differentiate into mesoderm and endoderm as compared to EBs induced from wild type hESCs, particularly in. The expression of the genes in control cells was arbitrary considered "1". All data were presented as mean ± SD (n=3).

Figure S2. Loss of PATZ1 globally activates human ES cell differentiation. (A) RT-qPCR validation for RNA-seq results. *β-ACTIN* serves as endogenous controls for quantification. (B-C) RNA-seq heatmap generated from pluripotency (B) and development (C) related gene expression levels. Relatively highly expressed genes were shown in dark blue, while low expressed genes were colored pale green. All data were presented as mean ± SD (n=3).

Figure S3. PATZ1 binds to distal promoters of *OCT4* loci. (A/B) Top: CR3/CR4 region was cloned into the downstream of luciferase gene driven by SV40 promoter. Bottom: Luciferase activity of CR3/CR4 in PATZ1-depleted cells.

Figure S4. Genome-wide location analysis of PATZ1. (A) RT-PCR validation for ChIP-Seq. (B) Left: Genomic distribution of PATZ1 binding loci by statistics of ChIP annotation. Right: ngsPlot depicted the distribution of average reads of PATZ1, which were highly enriched in TSS. (C) On average, approximately 500 bp of PATZ1 binding peaks were distributed in a 50-percentile position of summits surrounding the PATZ1 motif. (D) Endogenous Co-IP of PATZ1 and OCT4 and NANOG confirmed the interaction of PATZ1/OCT4 and PATZ1/NANOG. The input fraction was total hESC lysate while IgG IP served as negative control. (E) GO analysis of PATZ1-associated genes (biological process). (F) RNA-editing results indicated that the occurrence of differential A→G editing events upon *PATZ1* knockdown. High frequency of pluripotency gene, *DPPA4*, *LIN2A* and *NANOG* underwent RNA-editing upon *PATZ1* knockdown. All data were presented as mean ± SD (n=3).

Figure S5. Loss of PATZ1 impairs cell proliferation and triggers cell apoptosis. (A) Cell viability of hESCs at 24- and 48-hours after transfection of *PATZ1*-RNAi. (B)

Wright-Giemsa staining showed the morphology of hESCs upon *PATZ1*-RNAi. The arrows indicated the shrinking chromosome plates. Scale bar=100 μm. (C) mRNA level of cell cycle genes upon *PATZ1* knockdown. (D) Summary of each cell population labeled with the fluorescence intensity of Annexin $V^{+}PI^{-}$. (E) Summary of each cell generation labeled with the fluorescence intensity of CSFE.Right: The summary of each cell cycle labeled with the fluorescence intensity of PI. (F) Statistics of each cell population labeled with the fluorescence intensity of PI. (G) RNA-seq heatmap generated from cell cycle phases gene expression levels. Relatively highly expressed genes were shown in dark purple, while low expressed genes were colored in pale purple. All data were presented as mean ± SD (n=3). Significance: * p <≤ 0.05, ** $p \leq 0.01$, *** $p \leq 0.001$.

Figure S6. Absence of PATZ1 destroys the mitochondrial function. (A) A representative graph for Seahorse measurements of ECR. (B) Quantification of ECR parameters. (C-D) Knockdown of PATZ1 did not reduce the transcriptomic (C) and protein level (D) of mitochondria biogenesis genes: MFF, DRP1, and TOM20. GAPDH served as loading control. (E) Loss of PATZ1 significantly reduced the JC-red fluorescence (polarization, high membrane potential), while increased the JC-green fluorescence. Scale bar=75 μm. (F) Quantification of mitochondrial DNA copy number, *MT-ND1* and *MT-ND4* relative to nuclear DNA measured qPCR upon PATZ1 depletion and normalized to *B2M*. (G) Protein sequence alignment of PATZ1 from different mammals. The reactivity of cysteines is enhanced by positively charged amino acids like arginine (R) and lysine (K), as indicated those amino acids with a '+" sign.

Figure S7. PATZ1 regulates homeostasis of hESCs. (A) knockdown of *PATZ1* caused the misexpression of metabolic genes, including genes involved in glycolysis, fatty acid oxidation, tricarboxylic acid cycle, lipid metabolism, and amino acid biosynthesis. (B) Depletion of PATZ1 reduced the protein level of representative glycolytic genes: GLUT1, PDH, G6PD, LDHA, HK1, HK2 and PKM2. GAPDH served as loading control. (C) Snapshots of PATZ1 ChIP-seq signals at metabolic genes loci: *PFKP*, *PHGDH*, *ELOVL5*, *GLUD1*, *PTGER1*, and *PKM*. (D) Heatmap of metabolite levels, as measured by LC/MS/MS metabolomics in hESCs upon *PATZ1* KD.

Supplementary figures

Figure S1. PATZ1 is essential for hESC pluripotency.
Figure S2. Loss of PATZ1 globally activates human ES cell differentiation.
Figure S3. PATZ1 binds to distal promoters of *OCT4* loci.
Figure S4. Genome-wide location analysis of PATZ1.

Figure S5. Loss of PATZ1 impairs cell proliferation and triggers cell apoptosis.
Figure S6. Absence of PATZ1 destroys the mitochondrial function.
Figure S7. PATZ1 regulates homeostasis of hESCs.

Supplementary Tables

Supplementary Table 1. Primers for plasmid construction.
Supplementary Table 2. RT-PCR primers being used.
Supplementary Table 3. Primary antibodies being used.
Supplementary Table 4. Top 100-ranked peak heights of PATZ1-ChIP peaks.
Supplementary Table 5. Differential expression analysis of altered gene in RNA-seq analysis upon *PATZ1* RNAi.
Supplementary Table 6. Calculation equations of ATP production from Seahorse assay.
Supplementary Table 7. Quantification of metabolites by LC/MS/MS.

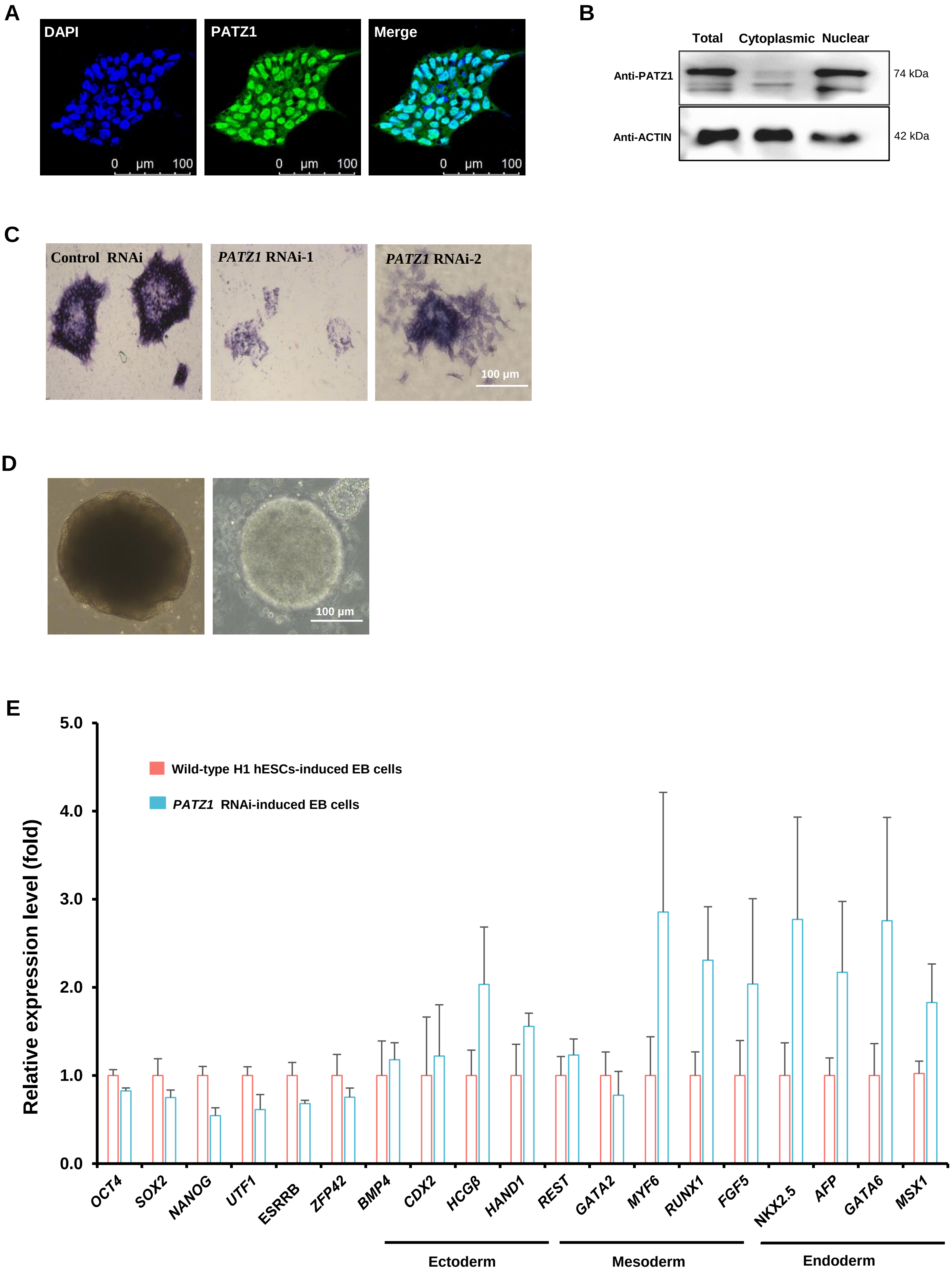

A
DAPI
PATZ1
Merge
0 μm 100
B
Total
Cytoplasmic
Nuclear
Anti-PATZ1
Anti-ACTIN
74 kDa
42 kDa
C
Control RNAi
PATZ1 RNAi-1
PATZ1 RNAi-2
100 μm
D
100 μm
E
Wild-type H1 hESCs-induced EB cells
PATZ1 RNAi-induced EB cells
Relative expression level (fold)
5.0
4.0
3.0
2.0
1.0
0.0
OCT4
SOX2
NANOG
UTF1
ESRRB
ZFP42
BMP4
CDX2
HCGβ
HAND1
REST
GATA2
MYF6
RUNX1
FGF5
NKX2.5
AFP
GATA6
MSX1
Ectoderm
Mesoderm
Endoderm

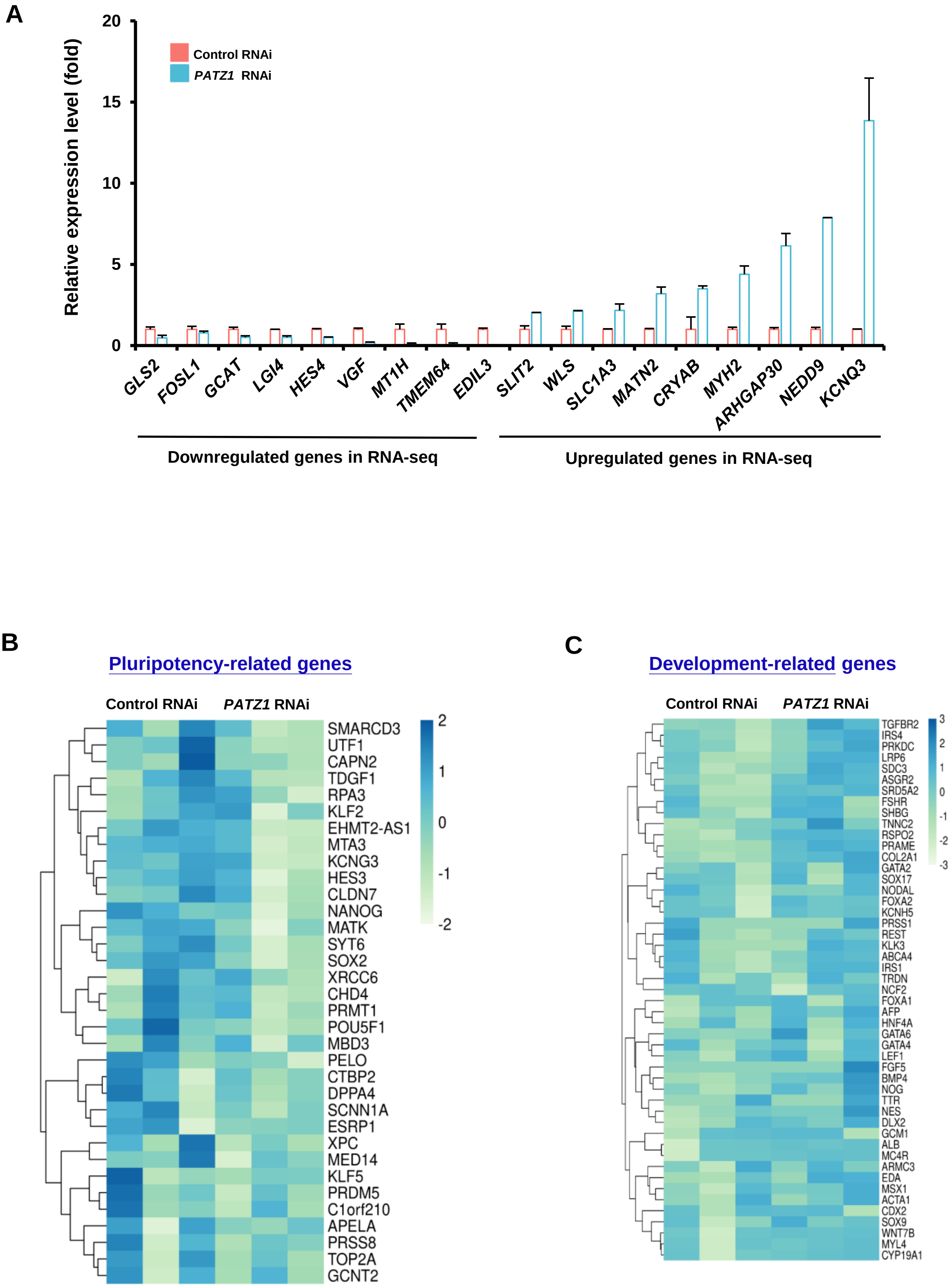
A
Relative expression level (fold)
20
15
10
5
0
Control RNAi
PATZ1 RNAi
GLS2
FOSL1
GCAT
LGI4
HES4
VGF
MT1H
TMEM64
EDIL3
SLIT2
WLS
SLC1A3
MATN2
CRYAB
MYH2
ARHGAP30
NEDD9
KCNQ3
Downregulated genes in RNA-seq
Upregulated genes in RNA-seq
B
Pluripotency-related genes
Control RNAi
PATZ1 RNAi
SMARCD3
UTF1
CAPN2
TDGF1
RPA3
KLF2
EHMT2-AS1
MTA3
KCNG3
HES3
CLDN7
NANOG
MATK
SYT6
SOX2
XRCC6
CHD4
PRMT1
POU5F1
MBD3
PELO
CTBP2
DPPA4
SCNN1A
ESRP1
XPC
MED14
KLF5
PRDM5
C1orf210
APELA
PRSS8
TOP2A
GCNT2
2
1
0
-1
-2
C
Development-related genes
Control RNAi
PATZ1 RNAi
TGFBR2
IRS4
PRKDC
LRP6
SDC3
ASGR2
SRD5A2
FSHR
SHBG
TNNC2
RSPO2
PRAME
COL2A1
GATA2
SOX17
NODAL
FOXA2
KCNH5
PRSS1
REST
KLK3
ABCA4
IRS1
TRDN
NCF2
FOXA1
AFP
HNF4A
GATA6
GATA4
LEF1
FGF5
BMP4
NOG
TTR
NES
DLX2
GCM1
ALB
MC4R
ARMC3
EDA
MSX1
ACTA1
CDX2
SOX9
WNT7B
MYL4
CYP19A1
3
2
1
0
-1
-2
-3

A

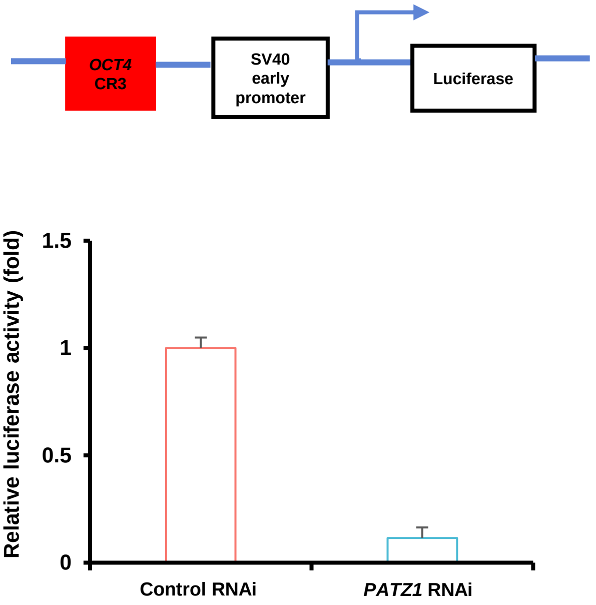

OCT4
CR3
SV40
early
promoter
Luciferase
Relative luciferase activity (fold)
1.5
1
0.5
0
Control RNAi
PATZ1 RNAi

B

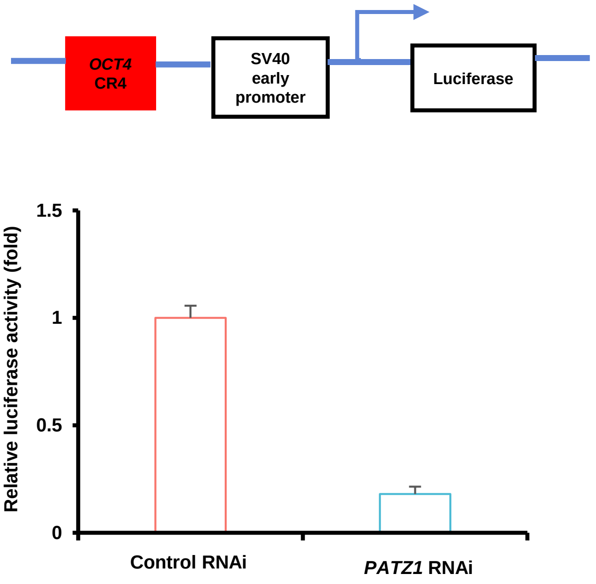

OCT4
CR4
SV40
early
promoter
Luciferase
Relative luciferase activity (fold)
1.5
1
0.5
0
Control RNAi
PATZ1 RNAi

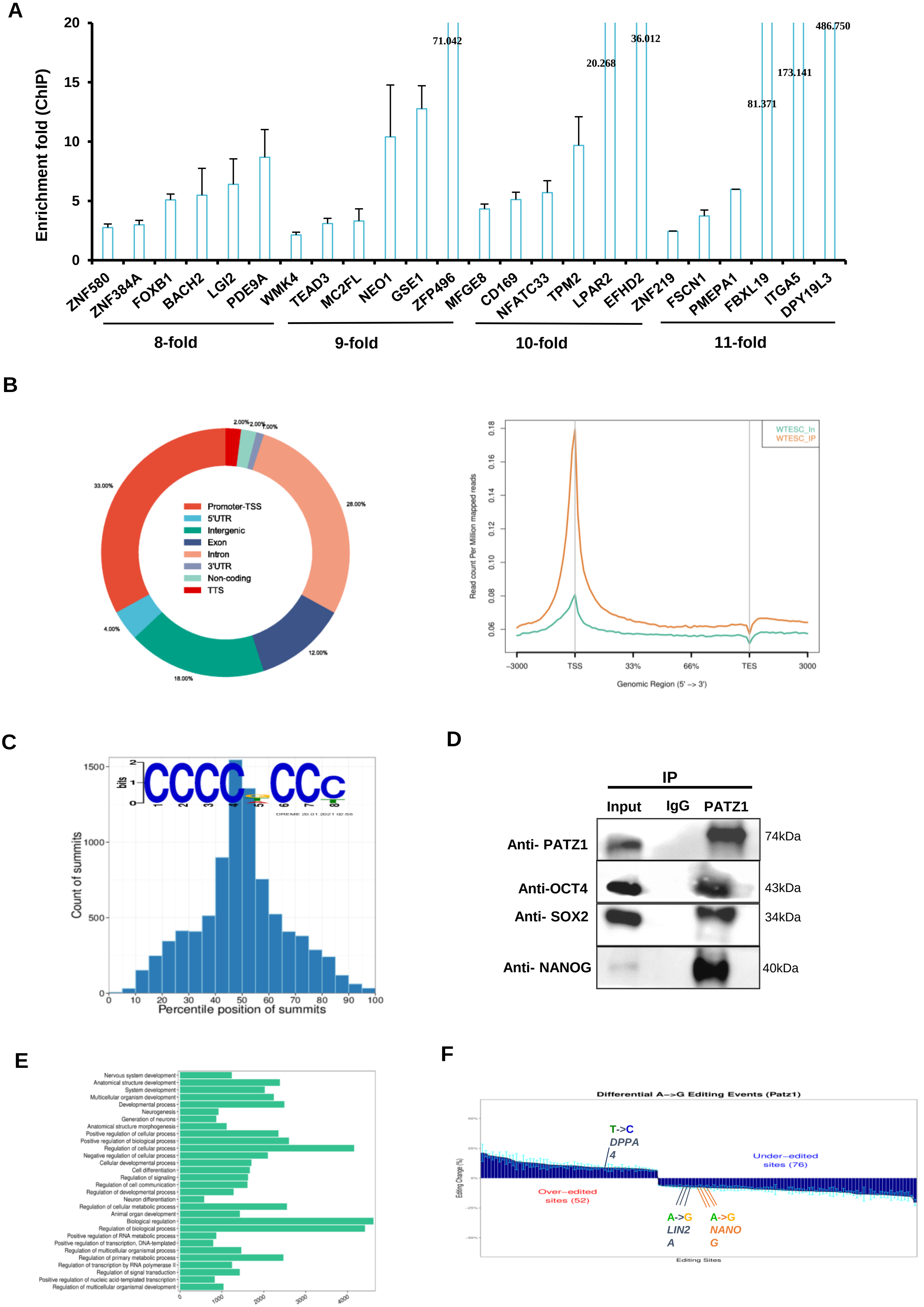

A
Enrichment fold (ChIP)
0
5
10
15
20
71.042
20.268
36.012
81.371
173.141
486.750
ZNF580
ZNF384A
FOXB1
BACH2
LGI2
PDE9A
WMK4
TEAD3
MC2FL
NEO1
GSE1
ZFP496
MFGE8
CD169
NFATC33
TPM2
LPAR2
EFHD2
ZNF219
FSCN1
PMEPA1
FBXL19
ITGA5
DPY19L3
8-fold
9-fold
10-fold
11-fold
B
2.00%
2.00%
1.00%
33.00%
28.00%
4.00%
12.00%
18.00%
Promoter-TSS
5'UTR
Intergenic
Exon
Intron
3'UTR
Non-coding
TTS
WTESC_In
WTESC_IP
Read count Per Million mapped reads
0.06
0.08
0.10
0.12
0.14
0.16
0.18
−3000
TSS
33%
66%
TES
3000
Genomic Region (5' −> 3')
C
Count of summits
Percentile position of summits
0
500
1000
1500
0
10
20
30
40
50
60
70
80
90
100
bits
CCCC CCc
D
IP
Input
IgG
PATZ1
Anti- PATZ1
Anti-OCT4
Anti- SOX2
Anti- NANOG
74kDa
43kDa
34kDa
40kDa
E
Nervous system development
Anatomical structure development
System development
Multicellular organism development
Developmental process
Neurogenesis
Generation of neurons
Anatomical structure morphogenesis
Positive regulation of cellular process
Positive regulation of biological process
Regulation of cellular process
Negative regulation of cellular process
Cellular developmental process
Cell differentiation
Regulation of signaling
Regulation of cell communication
Regulation of developmental process
Neuron differentiation
Regulation of cellular metabolic process
Animal organ development
Biological regulation
Regulation of biological process
Positive regulation of RNA metabolic process
Positive regulation of transcription, DNA-templated
Regulation of multicellular organismal process
Regulation of primary metabolic process
Regulation of transcription by RNA polymerase II
Regulation of signal transduction
Positive regulation of nucleic acid-templated transcription
Regulation of multicellular organismal development
0
1000
2000
3000
4000
F
Differential A−>G Editing Events (Patz1)
Editing Change (%)
50%
25%
0%
−25%
−50%
T->C
DPPA
4
Under−edited
sites (76)
Over−edited
sites (52)
A->G
LIN2
A
A->G
NANO
G
Editing Sites

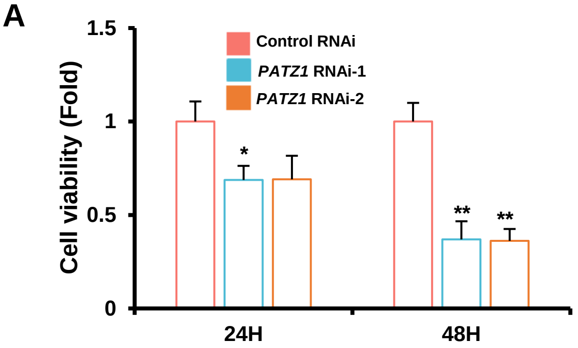

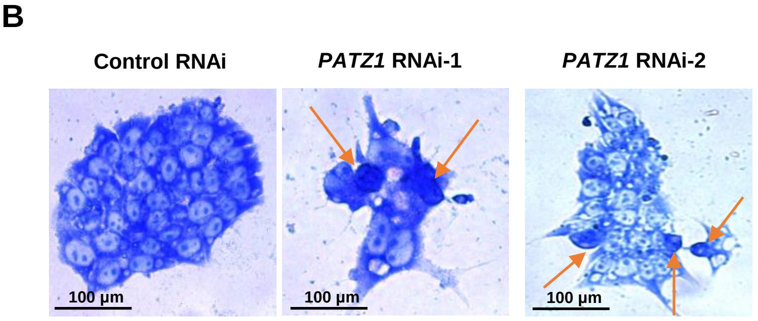

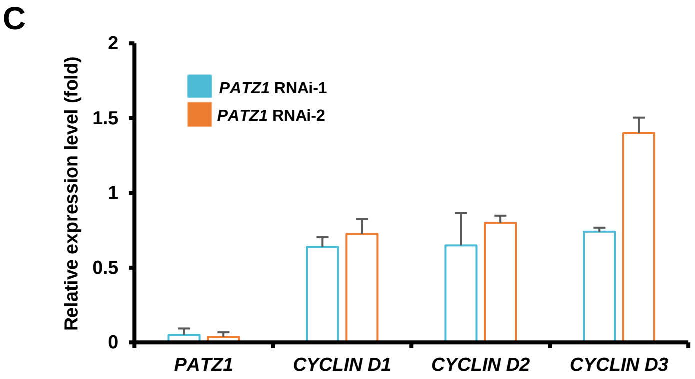

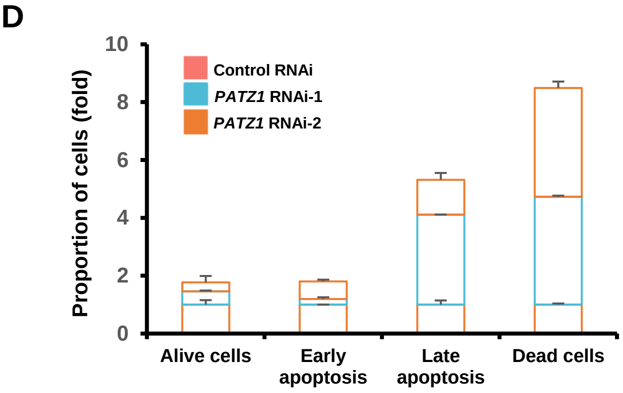

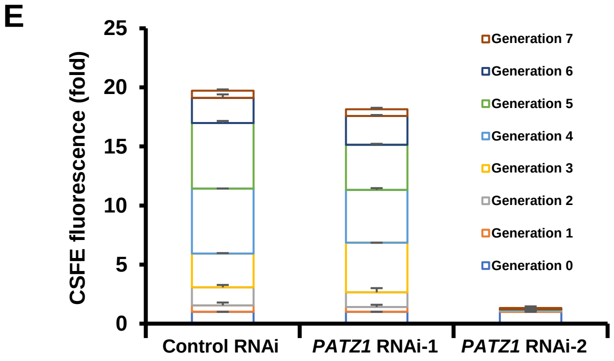

F

	Control RNAi	*PATZ1* RNAi-1	*PATZ1* RNAi-2
**Dip G1**	40.06 ± 1.92	42.10 ± 1.73	38.77 ± 2.51
**Dip S**	44.39 ± 1.09	41.68 ± 1.51*	39.27 ± 2.13*
**Dip G2**	15.55 ± 2.42	16.22 ± 0.86	21.96 ± 3.45*

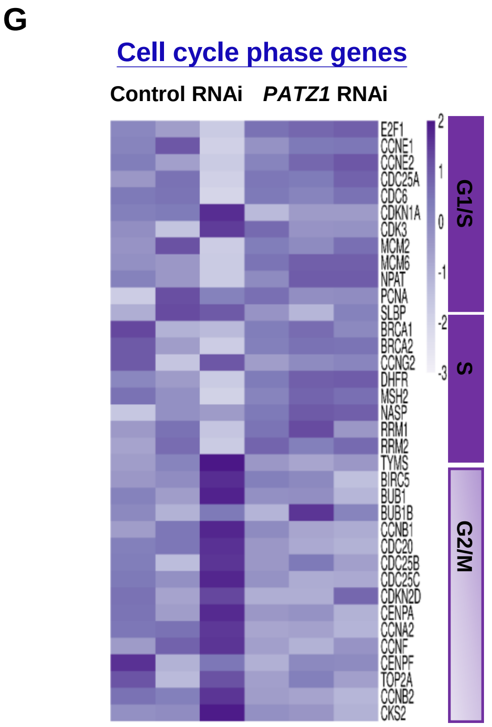

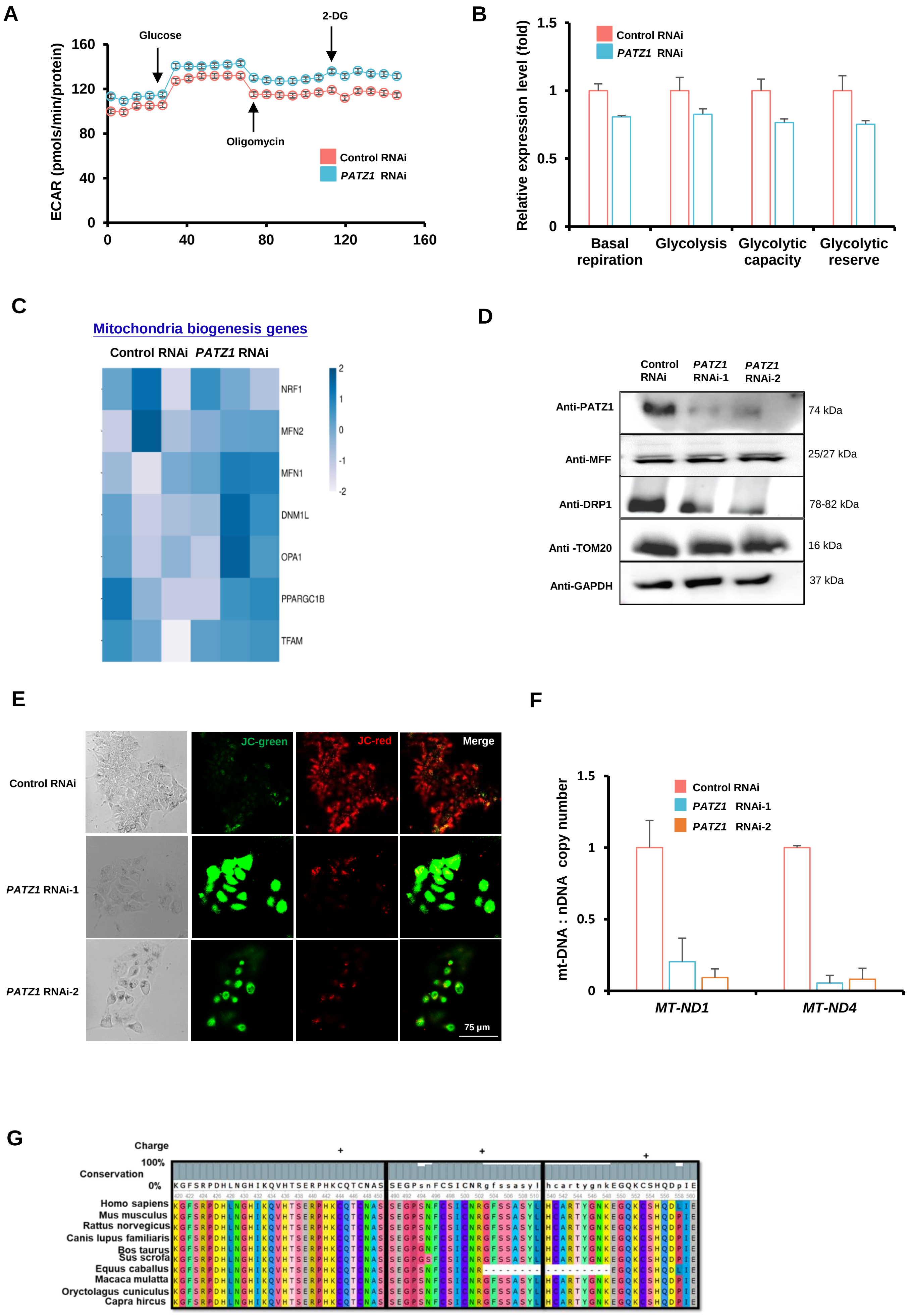
A
ECAR (pmols/min/protein)
Glucose
2-DG
Oligomycin
Control RNAi
PATZ1 RNAi
0
40
80
120
160
B
Relative expression level (fold)
Control RNAi
PATZ1 RNAi
1.5
1
0.5
0
Basal repiration
Glycolysis
Glycolytic capacity
Glycolytic reserve
C
Mitochondria biogenesis genes
Control RNAi PATZ1 RNAi
NRF1
MFN2
MFN1
DNM1L
OPA1
PPARGC1B
TFAM
2
1
0
-1
-2
D
Control RNAi
PATZ1 RNAi-1
PATZ1 RNAi-2
Anti-PATZ1
74 kDa
Anti-MFF
25/27 kDa
Anti-DRP1
78-82 kDa
Anti -TOM20
16 kDa
Anti-GAPDH
37 kDa
E
JC-green
JC-red
Merge
Control RNAi
PATZ1 RNAi-1
PATZ1 RNAi-2
75 µm
F
mt-DNA : nDNA copy number
Control RNAi
PATZ1 RNAi-1
PATZ1 RNAi-2
1.5
1
0.5
0
MT-ND1
MT-ND4
G
Charge
100%
Conservation
0%
Homo sapiens
Mus musculus
Rattus norvegicus
Canis lupus familiaris
Bos taurus
Sus scrofa
Equus caballus
Macaca mulatta
Oryctolagus cuniculus
Capra hircus

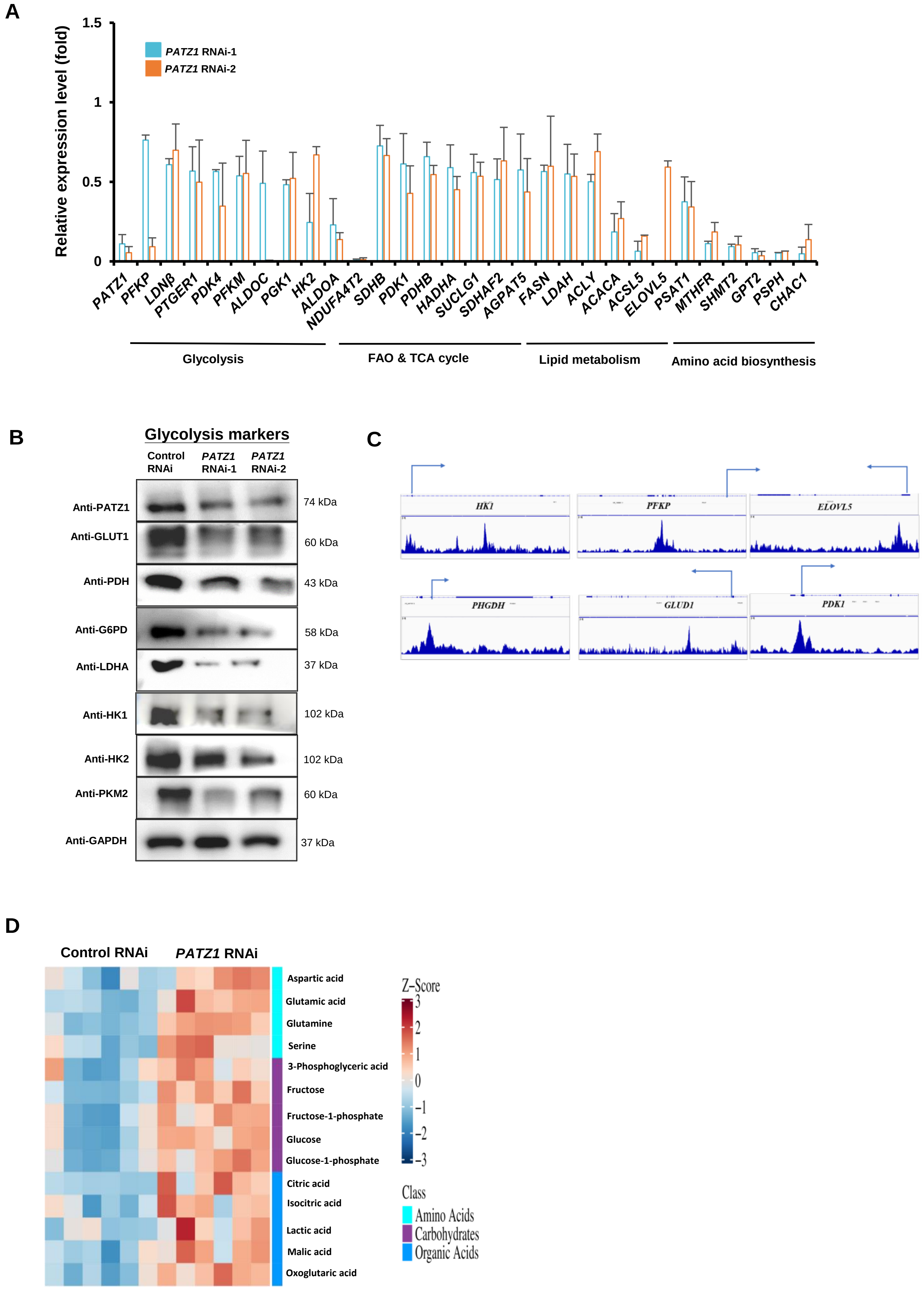

A
Relative expression level (fold)
1.5
1
0.5
0
PATZ1 RNAi-1
PATZ1 RNAi-2
PATZ1
PFKP
LDNβ
PTGER1
PDK4
PFKM
ALDOC
PGK1
HK2
ALDOA
NDUFA4T2
SDHB
PDK1
PDHB
HADHA
SUCLG1
SDHAF2
AGPAT5
FASN
LDAH
ACLY
ACACA
ACSL5
ELOVL5
PSAT1
MTHFR
SHMT2
GPT2
PSPH
CHAC1
Glycolysis
FAO & TCA cycle
Lipid metabolism
Amino acid biosynthesis
B
Glycolysis markers
Control RNAi
PATZ1 RNAi-1
PATZ1 RNAi-2
Anti-PATZ1
74 kDa
Anti-GLUT1
60 kDa
Anti-PDH
43 kDa
Anti-G6PD
58 kDa
Anti-LDHA
37 kDa
Anti-HK1
102 kDa
Anti-HK2
102 kDa
Anti-PKM2
60 kDa
Anti-GAPDH
37 kDa
C
HK1
PFKP
ELOVL5
PHGDH
GLUD1
PDK1
D
Control RNAi
PATZ1 RNAi
Aspartic acid
Glutamic acid
Glutamine
Serine
3-Phosphoglyceric acid
Fructose
Fructose-1-phosphate
Glucose
Glucose-1-phosphate
Citric acid
Isocitric acid
Lactic acid
Malic acid
Oxoglutaric acid
Z-Score
3
2
1
0
-1
-2
-3
Class
Amino Acids
Carbohydrates
Organic Acids

Supplementary Table 1. Primers for plasmid construction.

Description	Sequence
Negative control	GTTCTCCGAACGTGTCACGTT
PATZ1-shRNA-1	GCGCCGATATAATGCTCTTTC
PATZ1-shRNA-2	CGAGTACTTTGAGTCGGTGTT
OCT4 CR2-pSV40-Luc	AAAAAATATCTGACTTCAGGTTCAAAGAAG
p*NANOG* PP-Luc	GCTGGTTTCAAACTCCTGACTTCAG

Supplementary Table 2. RT-PCR primers being used.

Description	Forward (5' – 3')	Reverse (3' – 5')
PATZ1	GGCCTGATCACTTGAACGGA	GGCAGAGGAGAAACCTCGG
DPPA4	CTGGTGCCAACAATTGAAGCT	AGGCACACAGGCGCTTATATG
OCT4	GGCTCTCCCATGCATTCAAAC	CCAAAAACCCTGGCACAAACT
NANOG	GCATCCGACTGTAAAGAATCTTCA	CATCTCAGCAGAAGACATTTGCA
SOX2	TGCGAGCGCTGCACAT	GCAGCGTGTACTTATCCTTCTTCA
UTF1	CGCCGCTACAAGTTCCTTAAA	GGATCTGCTCGTCGAAGGG
ESRRB	CCTCAGAGATGCGCAGGTTA	CCTCCGTGGAATGGTCTGAG
KLF4	GTCTCTTCGTGCACCCACTT	CCAGCCCGAGCTACAAATCC
BMP4	TGGGATTCCCGTCCAAGCTAT	AGGAATCATGGTGTCTTGACAGA
CDX2	CTCGGCAGCCAAGTGAAAA	GGTCCGTGTACACCACTCGAT
HCGβ	ACGACTGAGTCTCTGAGGTCACTT	ACAGATGGTGGTGGTGACGGTGAT
GCM1	CACAGTGCTGTCTGCTTCTCC	CGACTCCCCTCAGAAATGCT
PAX6	CATACCAAGCGTGTCATCAATAAAC	TGCGCCCATCTGTTGCT
HAND1	GGGTTAAACAGGTCTTTGGGCT	CCCTATTAACGCCGCTCCAT
REST	GATCCCCTCCCCCGTTTTAG	AACTGCCACCCCAAATGGAA
NESTIN	CCAGAGGTCATGTTGGGGTC	CAAGGTGAAGGGGCATCACT
T	GTGACCAAGAACGGCAGGAGG	TGTTCCGATGAGCATAGGGGC
GATA2	CAGCAAGGCTCGTTCCTGTT	GGCTTGATGAGTGGTCGGT
MYF6	GGGCTCGTGATAACGGCTAA	AAGGCATCGAAGGCTACTCG
RUNX1	GGAAGTCAACCTCTGCTGCT	CGGACCACAGAGCACTTTCT
NKK2.5	CTTCAAGCCAGAGGCCTACG	CCGCCTCTGTCTTCTTCAGC
FGF5	CCGGAAATGTAGCCAGGAGA	ACAATCCCCTGAGACACAGC
FOXA1	GAAGACCGGCCAGCTAGAGG	TGTTGATGGAGAACGGGTGG
FOXA2	TTGCTGGTCGTTTGTTGTGG	GGAGGAGTAGCCCTCGG
GATA4	GGAAGGAGCCAGCCTAGCAG	CTATTGGGGGCAGAAGACGG
GATA6	AGCACCAATCCCGAGAACAG	GCGAGACTGACGCCTATGTA
SOX17	CGCCGAGTTGAGCAAGA	TTCAGCCGCTTCACCTG
GLS2	CCTGCGCTACAACAAGCTCT	GGAAAAATTACCTTCCGTGGC

FOSL1	CATGTTCCGAGACTTCGGGG	GTGTTGATGCTTGGCACCAG
GCAT	TCCGGGTACAGATCTCAGCA	TGGCTCCTGCCTGATTCTTC
LGI4	GCTGTCACTCTCACTCGTCA	GTGTAAGCGAGCGAAGTCCT
HES4	ATCCGCCCTAGGGGCTC	GAGGGTTTTGAGCTGAGCGA
VGF	GCACAAGGTGGTTTTCGTCC	ACCCGTTGATCAGCAGAAGG
MT1H	GCAAAGGGGCGTCAGAGAAGT	GGAATGTAGCAAATGAGTCGGAGTT
TMEM64	TGGGCATGGGTCTGATGATG	GCAGGGTGGTACCCAAGTAA
EDIL3	GGATCATGAAGCGCTCGGTA	CAGGGACCAACCTCCACAAC
SLIT2	TGTCTGCAGTGATGAGGAAGA	AGCTCCAGGAGGGATGACTT
WLS	CACAACGGCAGTGTCCTACA	GGGGAGGGGAATGTGAACAG
SLC1A3	ACCCCAAGCATTCTGTGCAT	CCAGTCCACACTGAGTGCAT
MATN2	GGATGGGAAGAGGTGTGTGG	ATCCTTCATGGCACTGGCAA
CRYAB	CAAACGGCCTGGGTGGATA	TTGTGATCCGGGATTTGGCA
MYH2	GCCTTTCAAGAGGGACACTG	TCAAAGGGCCTATTCTGGGC
ARHGAP30	CTGGTAGGGCACAGGTCTTG	TTGGAGGAGACCCCTGAGAG
NEDD9	CCTCTAGAAGCAAGTCCGCTC	GAGGTGACAGCTAGTCCTGC
KCNQ3	GCTGCCCTAAACTTCCACAA	TGAAAAGCCACCACTAGCCA
p2	GGACTCGGAAGAGGTTCACCTTCGG	GTCGCCTCCGCTTGCTGAACTCAATGC
OCT4 amplicon 1	GGCCAGAAATACACCACCTACAGTACAAAT	CAGTGTTTCTCAGCTTGCCATCCTC
OCT4 amplicon 2	CCACCCCCACCAGGCCCCATAATCTACC	GTTGCCTCTGTTCGTGTGCCCATCTCCTGT
OCT4 amplicon 3	AGAAGTGAACACAGCTGCAACCCCACTG	TCTTCATCTTGGTGGCATCCGTGAGTCTTT
OCT4 amplicon 4	GGCAGTCTACTCTTGAAGATGGGGTGAAAT	CTATCCCTCAATGCATCAACAGCCATAA
NANOG amplicon 1	GTGCTGATTAAGAGAGACAGGAGGGCAAGT	GTGAAACAAGGAAATGAAATTGGGCGTAA
NANOG amplicon 2	CAATACTTTTCGAGTCTTTGCATTGTGAAC	CCCAAACCCAACATTCAAGAAACCTA
NANOG amplicon 3	GATTTAAAAGTTGGAAACGTGGTGAACCTA	CCTTGTGAATTCTCAGTTAATCCCGTCT
NANOG amplicon 4	TTAGCCAGAAGACCTAACGTCATAGCTCAG	CCCAGTAACATCCACAAACCCAGAGA
ZNF580	GGGTTCCCATAAACAAGGGGA	TCCCTCTCCCTGTTACACACA
ZNF385A	AGGAACAAAGGCAAACCGAGG	ACAGGAAGGGATCCTGGAGAC

FOXB1	GGCCGGATCTCTTTACGTCT	CTTTCCTAACTCCCAGCGCC
BACH2	AGTTCGTGGGTCACCGAAAG	TAGCGATTGAGAGTGTGGGC
LGI2	ATCCGATGCCTGCAATACCT	ATCAAGCCGGTTCTCCACAC
PDE9A	CTGGACATCGATGGACGCAT	TGTGCCAGCCCTTGAATGTA
WNK4	GTAACTGTGGCCAAGGGGAG	GGCCAGAGTGGCTGTTTTTG
TEAD3	TCAGGGCTGGAACTCTGAATC	CTGGGTTGTGGAGGGTGTC
MCF2L	GGTTTCCAAGCACACGGGTA	AGAAAGGAACACGAGGGCG
NEO1	GCCGAAGAGAAAGGGGATCG	GAACCTGCCTCCAGGTACAA
GSE1	CGGCCAGTGCGGGTATAAAT	GCAGACCACAAACGAAAAGGG
ZNF496	CTTCCTGCTCCGCTCTAGGA	CCAGAGAAGGAGAACAGCGG
MFGE8	TTCTCCTCTGGGACCGGAAT	AAGAGGCAGATATCGCGGAC
CD169	GGAGAAAGCTAAGGCTCGCA	CCCGTAGGATTCCCGTTGG
NFATC3	ACTCAACTTCCAGCAAGGGAC	CTCAGAGTTACCTGGACGCC
LPAR2	CGAAAATTCCCGCCGTGACC	GAGCCAAGTCGGATCAGGGA
EFHD2	ACCCTCATTGAGAACGCCAG	CTCTTCGTGCCCACTGTAGC
ZNF219	TGGGAAAATCCCCCTCCCAG	GCGGCTGTAACTCAGCTTTT
FSCN1	GAGACAACCTAGTTGGGCGT	CAAGGACGAGCGAGGTTTGA
PMEPA1	CAAGGAGATCGGGTTTCGCT	CCTAGCTCTGGCCCTTTAAGA
FBXL19	GGACACGTGTGAGGTGGGTT	GGCTGGAGATGAGTTTGGAGTA
ITGA5	GGCCGGTAAAACTCCACTGA	GCGTTGAGTCATTCGCCTCT
DPY19L3	ACAGACCGGACTCAATGCG	CGCGGAGCTTTGTGGAAATG
BNIP3	CCACCCTCAGCATGAGGAAC	TGGCCACCCCAGGATCTAAC
HK1	CGCAGCTCCTGGCCTATTA	CTTCCACTCCGCTCGCTTTA
PKM	CCCGATCAGTGGAGCTGAAG	AGGAAGTCGGCACCTTTCTG
PFKP	GTCACACCGCTTCCCTTGTC	TTCTGCACGGCCTCCTTATC
LDNβ	CCTGGTAGGTTTCGGCTCAG	CAAGGACAAGTAGGGCCTGG
PTGER1	GTACATCCTACTGCGCCAGG	GGGCCTCTGGTTGTGCTTAG
PDK4	TTCCCGGAATGCTCCTTTGG	GCACTGGTGTAGACCCACTT
PFKM	GGCGGAGGAGAGCTAAGACTA	TACCAACTCGAACCACAGCC
ALDOC	CAGCCTCATCTGTTTGCGGA	ATGGTGACAGCTCCCTGTGC
PGK1	ACCGAATCACCGACCTCTCT	TGGGACAGCAGCCTTAATCC
HK2	GTGAATCGGAGAGGTCCCAC	CAAGCAGATGCGAGGCAATC
ALDOA	TCCCCATCAATAGGGCCGAC	CCACATGTGTCCCCGATCTT
NDUFA4L2	GTGTGTTGCCATCGCGTCT	GGCTCCTGCCATATCGTTGT
IDH3B	TACGCAGTCTTTGAGACGGG	GGCGTGAAGGTGAAGCTGAT
ECH1	TACTGACCCGGCGACTGA	CGAAGCCATGTCCATCAGGT
MLYCD	CTTCGTGACAGACGGTGTGA	ACTCTGGAGGCAGCAGGATA
PDHA2	CGAGTTGCCCAGAAATCAGC	GGTGACAGAAACCGCGAATG

SDHB	CACTCTAGCTTGCACCCGAA	CCAGCGATAGGCCTGCATAA
PDK1	AAGCAGTTCCTGGACTTCGG	TCTTGCAGGCCATACAGCAT
PDHB	AGAGGCGCTTTCACTGGAC	ATTCACAAATGGGCCGCAAC
HADHA	GGTTTGGAGGTGGAAACCCA	CAGGCGGAACTGGATGTCTT
SUCLG1	TCTTTGTGCGTTGGCATTGG	CCGGCATGACCCATTCTTCT
SDHAF2	GATGCTTGCTCTGTCAAGGC	TAGGCTCGTTAATCAGGCGG
CPTIC	GATGCTTGCTCTGTCAAGGC	AAGGACGTCAATTCGGGACC
DGAT2	GCTTCAGCCATGAAGACCCT	CCACTCCCCATCTCCCAGA
G2E3	TTTGAATCCTGTGGGCCGTT	AACACAAGCAAGGAGAGCTGG
AGPAT5	GCGCTACGTGCTGAAAGAAG	AGCAAATGCCTGACTAGCTGA
FASN	CCTGGCTGCCTACTACATCG	CACATTTCAAAGGCCACGCA
LDAH	GGTGAGCGTAAAAGTCTGGATTC	TGGGTGACTCAGACATTCGT
ACLY	GGACTTCGGCAGAGGTAGAG	AGGAGTTCTTTGCCCGTCTG
ACACA	CGAGGACTGGGAGGATGGG	GTTATCCCCAAACCCAGGCA
ACSL5	GTACACGGGGAGAGCTTACG	GGCTTTGACTTGGTTTTGGCA
ELOVL5	TCCAAAAGGGAGCAACCCC	ATCTGATTCTCCTGCGGTGC
ASNS	CGCCCAGATTTTCTTCAATCACA	TTCCAAACAGCGGGTCAACT
PHGDH	AACCGCAGCTTCTTGGCTTA	ACTGATGAGCACTTTCCGCA
MTHFD2	AGAGCTTTGGAGAAACCAGCC	CCTAGCTTGGCACAACCTGT
PSAT1	AAAAACAATGGAGGTGCCGC	ATGCCTCCCACAGACACGTA
GLUD1	TTCTTGGGCTTTTGTATCCGTT	ATAGTGCCCTATGGTGCTGG
MTHFR	CGCTAGGAATATGGGCCTCG	CTTTGGAGCTCTCACTGCCA
SHMT2	TACACAGCCCTTCTGCAACC	TCAGTGCCAGGTTGAGCTTAT
GPT2	CAAGCCTGGCTAAAACGAGA	TAGGGCAGACACACAACGTG
PSPH	AGAAAGCTGCAGGACTGGTG	AAAGGGAGTCCGCTCAAAGG
CHAC1	AGTGCAAGGGGAGCAGAAC	TCTTGTGCACTGGAGTGGGT

Supplementary Table 3. Primary antibodies being used.

**Primary antibodies**		
**Protein specificity**	**Source**	**Identifier**
Anti-β-ACTIN	Santa Cruz Biotechnology	sc-47778
Anti-GAPDH	Santa Cruz Biotechnology	sc-47724
Anti-PATZ1	Santa Cruz Biotechnology	sc-292109
Anti-OCT-3/4	Santa Cruz Biotechnology	sc-5279
Anti-NANOG	Santa Cruz Biotechnology	sc-134218
Anti-SOX2	Santa Cruz Biotechnology	sc-365823
Anti-SMAD2/3	Cell Signaling Technology	8685S
Anti-LIN28	Santa Cruz Biotechnology	sc-67266

Anti-GATA4	Santa Cruz Biotechnology	sc-25310
Anti-GATA6	Santa Cruz Biotechnology	sc-518050
Anti-SOX17	Santa Cruz Biotechnology	sc-130295
Anti-Rabbit IgG	Cell Signaling Technology	2729
Anti-Mouse IgG1	Cell Signaling Technology	5415S
Anti-Ki-67	Cell Signaling Technology	9449S
Anti-BAX	Cell Signaling Technology	5023S
PARP	Cell Signaling Technology	9532S
Anti-Cleaved PARP	Cell Signaling Technology	5625S
Anti-CYCLIN A1	Santa Cruz Biotechnology	sc-53233
Anti-CYCLIN B1	Cell Signaling Technology	12231S
Anti-GLUT1	Cell Signaling Technology	12939S
Anti-PDH	Cell Signaling Technology	2784S
Anti-G6PD	Cell Signaling Technology	12263S
Anti-LDHA	Cell Signaling Technology	3582S
Anti-HK1	Cell Signaling Technology	2024S
Anti-HK2	Cell Signaling Technology	2867S
Anti-PKM2	Cell Signaling Technology	4053S
Anti-MFF	Cell Signaling Technology	84580S
Anti-DRP1	Cell Signaling Technology	8570S
Anti-TOM20	Cell Signaling Technology	42406S
**Secondary antibodies**		
**Protein specificity**	**Source**	**Identifier**
Anti-Mouse HRP-linked	Cell Signaling Technology	7076P2
Anti-rabbit IgG, HRP-linked	Cell Signaling Technology	7074P2
Anti-Mouse IgG Alexa Fluor 488	Invitrogen	A-11001
Anti-Rabbit IgG Alexa Fluor 488	Invitrogen	A-11008
Anti-Rabbit IgG Alexa Fluor 555	Invitrogen	A-21429
Anti-Mouse IgG Alexa Fluor 555	Invitrogen	A-21422
Anti-Mouse IgG Alexa Fluor 647	Invitrogen	A-21235

Anti-Rabbit IgG Alexa Fluor 647	Invitrogen	A-21245

Supplementary Table 4. Top 100-ranked peak heights of PATZ1-ChIP peaks.

**Top 100-ranked peak heights of PATZ1-ChIP peaks in human ESCs**									
**Chr**	**Start**	**End**	**Fold Change**	**P-vale ($-\log_{10}$)**	**Annotation**	**Detailed Annotation**	**Distance to TSS**	**Nearest Refseq**	**Gene Name**
chr10	1.25E+08	1.25E+08	50.1487	391.253	intron (NM_001290214, intron 1 of 10)	CpG	6666	NM_001329	CTBP2
chr5	1.4E+08	1.4E+08	40.0777	302.103	Intergenic	CpG	-7660	NM_032289	PSD2
chr22	31345384	31347350	36.1356	262.354	promoter-TSS (NM_032052)	promoter-TSS (NM_032052)	-21	NM_014323	PATZ1
chr22	38397923	38399514	36.024	254.13	5' UTR (NM_001289912, exon 1 of 15)	5' UTR (NM_001289912, exon 1 of 15)	197	NM_001289912	TPTEP2-CSNK1E
chr19	55283443	55284885	33.5169	228.832	5' UTR (NM_032430, exon 1 of 19)	5' UTR (NM_032430, exon 1 of 19)	167	NM_032430	BRSK1
chr11	1.18E+08	1.18E+08	33.496	337.369	promoter-TSS (NM_001197104)	promoter-TSS (NM_001197104)	-9	NM_005933	KMT2A
chr1	27234192	27235387	31.1218	283.319	5' UTR (NM_015023, exon 1 of 16)	5' UTR (NM_015023, exon 1 of 16)	157	NM_015023	WDTC1

chr21	8257854	8260823	27.8883	3056.2	non-coding (NR_003287, exon 2 of 3)	non-coding (NR_003287, exon 2 of 3)	2557	NR_146120	RNA5-8SN4
chr4	76949093	76950171	27.0524	141.569	promoter-TSS (NM_018243)	promoter-TSS (NM_018243)	-120	NM_018243	SEPTIN11
chr21	8440898	8447105	25.7497	7292.23	non-coding (NR_046235, exon 1 of 1)	non-coding (NR_046235, exon 1 of 1)	2855	NR_003287	RNA28SN5
chr7	1.43E+08	1.43E+08	25.4178	153.869	promoter-TSS (NM_001362783)	promoter-TSS (NM_001362783)	78	NR_161353	LOC100507507
chr9	1.21E+08	1.21E+08	25.4178	153.869	intron (NM_001286840, intron 1 of 14)	CpG	346	NM_015651	PHF19
chr15	89655133	89655764	24.4737	152.016	promoter-TSS (NM_198525)	promoter-TSS (NM_198525)	19	NM_198525	KIF7
chr8	1823147	1824494	24.342	126.73	promoter-TSS (NM_014629).2	promoter-TSS (NM_014629).2	-106	NM_014629	ARHGEF10
chr13	1.01E+08	1.01E+08	23.5287	137.757	promoter-TSS (NM_001286453)	promoter-TSS (NM_001286453)	-131	NM_032813	TMTC4
chr15	32899123	32899751	23.1999	214.139	intron (NM_001277313, intron 14 of 20)	intron (NM_001277313, intron 14 of 20)	168737	NM_198500	FMN1
chr18	3012267	3013109	22.9201	113.616	promoter-TSS (NM_014646)	promoter-TSS (NM_014646)	-741	NM_014646	LPIN2

chr5	90409652	90410242	22.5739	105.362	promoter-TSS (NM_004365)	promoter-TSS (NM_004365)	-53	NR_105024	LOC731157
chr11	69702079	69703392	21.9353	119.247	intron (NM_005117, intron 2 of 2)	CpG	1287	NM_005117	FGF19
chr19	4580186	4582214	21.8248	134.448	Intergenic	CpG	-21516	NM_020241	SEMA6B
chr17	77372758	77373726	21.5241	120.799	promoter-TSS (NM_001113493)	promoter-TSS (NM_001113493)	-95	NM_006640	SEPTIN9
chr11	47399595	47400791	21.3519	119.393	promoter-TSS (NR_039707)	promoter-TSS (NR_039707)	-777	NR_039707	MIR4487
chr15	83066823	83067592	21.3421	142.89	promoter-TSS (NM_025238)	promoter-TSS (NM_025238)	45	NM_025238	BTBD1
chr1	54487769	54489791	20.817	136.604	Intergenic	Intergenic	-59448	NM_015547	ACOT11
chr17	7627418	7628237	20.808	109.776	promoter-TSS (NM_001289115)	promoter-TSS (NM_001289115)	-6	NM_133491	SAT2
chr11	1.17E+08	1.17E+08	20.773	137.351	promoter-TSS (NM_025164)	promoter-TSS (NM_025164)	39	NM_025164	SIK3
chr1	1.55E+08	1.55E+08	20.6154	173.498	promoter-TSS (NM_017582)	promoter-TSS (NM_017582)	65	NM_017582	UBE2Q1
chr12	57127785	57129836	20.324	128.727	5' UTR (NM_002332, exon 1 of 89)	5' UTR (NM_002332, exon 1 of 89)	327	NM_002332	LRP1

chr2	85888552	85889471	20.2485	102.053	promoter-TSS (NM_001354227)	promoter-TSS (NM_001354227)	-21	NM_003896	ST3GAL5
chr19	40284693	40285598	20.2415	107.299	intron (NM_001626, intron 1 of 13)	CpG	200	NM_001626	AKT2
chr17	4498434	4499370	20.2363	102.897	promoter-TSS (NM_001124758)	promoter-TSS (NM_001124758)	21	NM_001124758	SPNS2
chr16	3879595	3881267	20.1726	117.651	promoter-TSS (NM_001079846)	promoter-TSS (NM_001079846)	282	NM_004380	CREBBP
chr14	68792886	68796453	20.0616	130.498	promoter-TSS (NM_004926)	promoter-TSS (NM_004926)	-755	NM_004926	ZFP36L1
chr19	47515089	47515686	20.0339	119.856	promoter-TSS (NR_038456)	promoter-TSS (NR_038456)	-324	NM_003827	NAPA
chr8	94640141	94641530	19.7233	117.13	promoter-TSS (NM_001034915)	promoter-TSS (NM_001034915)	-339	NM_017697	ESRP1
chr1	1.56E+08	1.56E+08	19.6166	177.954	promoter-TSS (NM_001282625)	promoter-TSS (NM_001282625)	705	NM_001093725	MEX3A
chr17	44123015	44124062	19.6093	115.901	5' UTR (NM_005474, exon 1 of 27)	5' UTR (NM_005474, exon 1 of 27)	103	NM_005474	HDAC5
chr11	46119696	46122598	19.5641	85.5172	promoter-TSS (NM_001352032)	promoter-TSS (NM_001352032)	-162	NM_016621	PHF21A
chr1	1.51E+08	1.51E+08	19.5637	136.634	intron (NM_001194938, intron 1 of 17)	CpG	533	NM_015100	POGZ

chr16	90018869	90020146	19.4068	104.041	promoter-TSS (NM_001286209)	promoter-TSS (NM_001286209)	-51	NM_024043	DBNDD1
chr17	67244464	67246436	19.3206	92.6227	promoter-TSS (NM_014877)	promoter-TSS (NM_014877)	-254	NM_014877	HELZ
chr17	67825086	67826346	19.2691	88.2085	5' UTR (NM_004459, exon 1 of 30)	5' UTR (NM_004459, exon 1 of 30)	213	NM_004459	BPTF
chr2	2.2E+08	2.2E+08	19.2574	113.065	TTS (NM_013335)	TTS (NM_013335)	-7128	NM_018674	ASIC4
chr16	70438737	70439612	19.1954	118.033	promoter-TSS (NM_006927)	promoter-TSS (NM_006927)	-74	NM_006927	ST3GAL2
chr2	2.36E+08	2.36E+08	19.0634	101.332	Intergenic	CpG	8868	NM_001485	GBX2
chr19	15448982	15450272	18.8227	97.2317	promoter-TSS (NR_031716)	promoter-TSS (NR_031716)	79	NR_031716	MIR1470
chr3	19146795	19148678	18.8067	89.557	promoter-TSS (NM_144633)	promoter-TSS (NM_144633)	-774	NM_144633	KCNH8
chr16	73032731	73033841	18.6142	101.71	intron (NM_006885, intron 1 of 9)	intron (NM_006885, intron 1 of 9)	14842	NM_006885	ZFHX3
chr2	43226180	43226980	18.5954	110.111	promoter-TSS (NR_027251)	promoter-TSS (NR_027251)	26	NM_006887	ZFP36L2
chr7	188280	189099	18.5449	98.1261	Intergenic	Intergenic	-3882	NM_020223	FAM20C

chr2	1.67E+08	1.67E+08	18.5191	92.9954	Intergenic	CpG	105432	NM_152381	XIRP2
chr18	3447545	3448721	18.4201	122.216	intron (NM_173207, intron 1 of 2)	CpG	591	NM_003244	TGIF1
chr17	43024690	43025646	18.3542	114.387	promoter-TSS (NM_005440)	promoter-TSS (NM_005440)	-63	NM_005440	RND2
chr11	1968980	1970196	18.3394	91.6548	Intergenic	Intergenic	20257	NR_132974	SNORD131
chr19	15199591	15201097	18.2749	90.6377	intron (NM_000435, intron 1 of 32)	CpG	651	NM_000435	NOTCH3
chr20	50931194	50932007	18.2741	77.301	promoter-TSS (NM_181442)	promoter-TSS (NM_181442)	-163	NM_015339	ADNP
chr9	1.37E+08	1.37E+08	18.2386	99.7428	Intergenic	Intergenic	29077	NR_135288	LINC01451
chr1	2.24E+08	2.24E+08	18.129	122.014	exon (NM_015176, exon 1 of 5)	exon (NM_015176, exon 1 of 5)	186	NM_015176	FBXO28
chr19	56120163	56121862	18.115	148.873	intron (NM_001002836, intron 1 of 2)	CpG	283	NM_001002836	ZNF787
chr3	1.77E+08	1.77E+08	18.0591	75.951	promoter-TSS (NM_001321193)	promoter-TSS (NM_001321193)	263	NM_024665	TBL1XR1
chr1	2314279	2315694	18.0329	93.288	Intergenic	CpG	37675	NM_001127577	LOC100129534

chr1	2.11E+08	2.11E+08	17.9031	146.274	promoter-TSS (NM_001136225)	promoter-TSS (NM_001136225)	-17	NM_018254	RCOR3
chr3	1.97E+08	1.97E+08	17.8974	101.198	promoter-TSS (NM_002577)	promoter-TSS (NM_002577)	45	NM_002577	PAK2
chr13	74133886	74135654	17.786	78.562	promoter-TSS (NM_007249)	promoter-TSS (NM_007249)	-841	NM_007249	KLF12
chr12	1.22E+08	1.22E+08	17.6905	117.093	intron (NR_152740, intron 2 of 3)	CpG	2777	NR_002809	LINC01089
chr10	68074796	68075721	17.6747	79.0973	promoter-TSS (NM_001278187)	promoter-TSS (NM_001278187)	25	NM_015601	HERC4
chr11	1.1E+08	1.1E+08	17.6517	74.137	promoter-TSS (NM_001260492)	promoter-TSS (NM_001260492)	-3	NM_002906	RDX
chr7	1.02E+08	1.02E+08	17.6202	86.3332	promoter-TSS (NM_181500)	promoter-TSS (NM_181500)	-191	NM_001913	CUX1
chr1	2.26E+08	2.26E+08	17.5551	129.004	5' UTR (NM_018212, exon 1 of 14)	5' UTR (NM_018212, exon 1 of 14)	253	NM_018212	ENAH
chr20	43390184	43390708	17.5524	78.7818	Intergenic	Intergenic	-67450	NM_006275	SRSF6
chr2	43100444	43101595	17.5177	89.3252	intron (NR_151714, intron 1 of 1)	CpG	1426	NR_151714	LINC02580
chr17	29567892	29568993	17.3855	84.8628	promoter-TSS (NM_001346079)	promoter-TSS (NM_001346079)	166	NM_138349	TP53I13

chr19	3061072	3063011	17.3391	87.9932	intron (NR_031672, intron 1 of 2).3	CpG-13254	454	NM_001130	TLE5
chr6	32968252	32969717	17.0737	102.64	5' UTR (NM_005104, exon 1 of 13).3	5' UTR (NM_005104, exon 1 of 13).3	390	NM_005104	BRD2
chr17	43300201	43300917	17.0674	220.495	promoter-TSS (NR_002716).10	promoter-TSS (NR_002716).10	-328	NR_002716	RNU2-1
chr16	70289346	70290044	17.0613	79.1089	promoter-TSS (NM_001257174)	promoter-TSS (NM_001257174)	-72	NM_007242	DDX19B
chr19	16075941	16077222	16.963	86.0938	promoter-TSS (NM_001367837)	promoter-TSS (NM_001367837)	86	NM_003290	TPM4
chr17	38450708	38451366	16.9552	110.343	intron (NM_001199417, intron 1 of 23).2	intron (NM_001199417, intron 1 of 23).2	22573	NM_020876	ARHGAP23
chr21	8214003	8216191	16.8092	2547.43	non-coding (NR_146144, exon 1 of 1)	non-coding (NR_146144, exon 1 of 1)	1209	NR_003287	RNA28SN5
chr14	1.05E+08	1.05E+08	16.8065	79.2225	promoter-TSS (NM_002226)	promoter-TSS (NM_002226)	-287	NM_002226	JAG2
chr14	99663944	99664627	16.7891	100.07	intron (NM_001329411, intron 7 of 8)	intron (NM_001329411, intron 7 of 8)	19175	NM_032425	HHIPL1

chr16	1151985	1154387	16.7741	79.9328	promoter-TSS (NM_021098)	promoter-TSS (NM_021098)	-55	NM_021098	CACNA1H
chr5	1.42E+08	1.42E+08	16.7726	91.9639	intron (NR_120664, intron 3 of 3)	CpG	31754	NR_120664	SPRY4-AS1
chr2	1.71E+08	1.71E+08	16.7692	67.9643	5' UTR (NM_012290, exon 1 of 21)	5' UTR (NM_012290, exon 1 of 21)	281	NM_012290	TLK1
chr19	3365682	3367871	16.6421	95.5616	intron (NM_001245002, intron 1 of 10)	CpG	193	NM_005597	NFIC
chr1	27527080	27528377	16.6349	122.859	Intergenic	Intergenic	-37561	NM_006990	WASF2
chr20	41137368	41138453	16.6238	83.5039	promoter-TSS (NR_109889)	promoter-TSS (NR_109889)	90	NR_109889	PLCG1-AS1
chr7	75878659	75879230	16.5101	96.728	promoter-TSS (NM_001346189)	promoter-TSS (NM_001346189)	-48	NM_020684	RHBDD2
chr14	1E+08	1E+08	16.4967	89.5039	Intergenic	CpG	8164	NR_106822	MIR6764
chr12	53220978	53223791	16.3845	97.9042	intron (NM_001243730, intron 2 of 8)	intron (NM_001243730, intron 2 of 8)	-2022	NM_000966	RARG
chr19	12838096	12839122	16.3568	81.9324	promoter-TSS (NM_014975)	promoter-TSS (NM_014975)	94	NM_014975	MAST1
chr12	53379976	53380870	16.3476	93.0083	promoter-TSS (NM_003109)	promoter-TSS (NM_003109)	-221	NM_003109	SP1

chr15	85794495	85795373	16.3376	74.7716	promoter-TSS (NM_022480)	promoter-TSS (NM_022480)	-9	NM_022480	KLHL25
chr1	2.06E+08	2.06E+08	16.2081	125.879	intron (NM_022731, intron 1 of 6)	CpG	582	NM_022731	NUCKS1
chr15	78872496	78873030	16.1577	76.6371	promoter-TSS (NM_001265605)	promoter-TSS (NM_001265605)	-18	NM_006791	MORF4L1
chr10	98030029	98031514	16.1075	76.4959	promoter-TSS (NM_018058)	promoter-TSS (NM_018058)	57	NM_018058	CRTAC1
chr13	27961301	27962166	16.1075	76.4959	3' UTR (NM_001354700, exon 3 of 3)	3' UTR (NM_001354700, exon 3 of 3)	7582	NM_001265	CDX2
chr19	49590798	49593601	16.1075	86.7087	intron (NM_020719, intron 1 of 13)	CpG	1017	NM_020719	PRR12
chr12	48851946	48852485	16.1041	85.126	promoter-TSS (NM_004818)	promoter-TSS (NM_004818)	-52	NM_004818	DDX23
chr20	43590387	43590999	16.0794	92.9202	promoter-TSS (NM_001303459)	promoter-TSS (NM_001303459)	-244	NM_016004	IFT52

Supplementary Table 5. Differential expression analysis of altered gene in RNA-seq analysis upon *PATZ1* RNAi.

**Differential expression analysis of pluripotency genes upon *PATZ1* RNAi**									
**Gene_ name**	**log2 fold change**	**pvalue**	**P adjust**	**Control RNAi fpkm**	**Control RNAi fpkm**	**Control RNAi fpkm**	***PATZ1* RNAi fpkm**	***PATZ1* RNAi fpkm**	***PATZ1* RNAi fpkm**
SMARCD3	-0.35969	0.250188	0.63102	2.939595	2.062871	3.522572	2.684312	1.80114	1.971093
UTF1	-0.80098	0.004318	0.089069	8.371304	9.381131	18.36595	8.074416	6.037973	6.198554
CAPN2	-0.23862	0.20836	0.590405	27.62578	25.42786	40.31131	26.78734	26.61334	24.21318
TDGF1	-0.04762	0.751399	0.920855	778.6861	840.0527	875.1391	832.8368	772.9164	772.3121
RPA3	-0.04595	0.783226	0.933135	69.89669	74.78333	81.45395	79.86254	71.03998	64.78331
KLF2	-0.09749	0.87472	0.965732	0.348949	0.516961	0.710488	0.767489	0.217694	0.452867
EHMT2-AS1	-0.52348	0.054936	0.341143	5.381228	7.349757	6.620706	6.178256	3.491222	3.601115
MTA3	-0.19728	0.229264	0.610356	76.30681	78.56499	79.42889	76.83918	60.84271	63.31992
KCNG3	-0.31523	0.240022	0.620856	1.836903	1.72813	2.105075	2.06265	1.170119	1.219327
HES3	-0.75418	0.231063	0.612289	31.34707	38.90582	52.09101	42.34224	10.25327	18.28609
CLDN7	-0.06293	0.685853	0.894616	98.69314	100.0887	109.0502	104.5929	90.13777	95.10179
NANOG	-0.29202	0.090015	0.426778	94.12503	87.0886	77.62782	79.02595	58.44372	70.05766
MATK	-0.29502	0.114716	0.473438	21.77245	23.43893	23.11367	19.51475	15.35111	19.9541
SYT6	-0.46904	0.023278	0.221885	14.68286	18.35614	20.4592	15.14181	10.24927	12.68945
SOX2	-0.19082	0.211998	0.594187	177.2632	191.5512	187.5786	169.1889	149.844	161.4132
XRCC6	-0.00635	0.969516	0.992003	386.6647	474.3803	437.1251	456.883	417.3649	403.5784
CHD4	-0.16385	0.39067	0.740412	12.57552	16.33665	14.17453	14.35984	11.61096	12.06791

PRMT1	-0.06876	0.684159	0.893922	553.0841	673.4699	610.8004	630.1578	533.6376	567.1663
POU5F1	-0.10908	0.477186	0.790893	2181.782	2431.669	2169.23	2131.052	2011.549	2065.9
MBD3	-0.08776	0.682236	0.893426	35.4579	50.96569	38.44159	44.83285	30.72153	40.88919
PELO	-0.14626	0.414475	0.755778	8.990931	8.707734	7.445133	7.839691	7.729228	6.803814
CTBP2	-0.06138	0.736697	0.915933	181.8853	155.8653	115.5049	152.563	132.3009	141.6068
DPPA4	-0.03595	0.814513	0.945914	758.0605	686.3243	594.6124	677.2144	622.9404	654.5651
SCNN1A	-0.16451	0.406332	0.750262	85.66466	95.82127	62.08351	76.07676	63.50068	75.17198
ESRP1	-0.00871	0.954628	0.988175	73.90409	74.83772	63.86782	69.15961	69.3621	69.83203
XPC	-0.27012	0.119979	0.481207	38.51684	30.58518	46.24368	28.89998	34.60178	30.23233
MED14	-0.15126	0.349008	0.712003	68.5876	67.32108	83.46328	56.75323	71.11884	66.68889
KLF5	-0.15237	0.658241	0.882567	2.146482	1.046043	1.229179	1.159184	1.363011	1.346428
PRDM5	-0.17632	0.296639	0.671648	25.33572	18.40743	20.09679	16.50247	20.62947	18.22352
C1orf210	-0.12921	0.541606	0.82551	11.01633	7.872519	8.347788	7.258819	9.417678	7.736131
APELA	-0.03224	0.849548	0.956202	86.32988	57.19952	86.10282	72.35694	69.55783	77.04358
PRSS8	-0.02351	0.866437	0.962947	121.4816	99.66324	114.4369	103.8034	111.3172	108.6974
TOP2A	-0.11914	0.443627	0.77055	454.7559	322.4182	457.8502	354.5155	402.8876	354.3603
GCNT2	-0.04296	0.778091	0.930828	62.21859	46.26366	59.04406	50.20665	58.32479	50.68604

DEA of developmental genes upon *PATZ1* RNAi

**Gene_ name**	**log2 fold change**	**pvalue**	**P adjust**	**Control RNAi fpkm**	**Control RNAi fpkm**	**Control RNAi fpkm**	***PATZ1* RNAi fpkm**	***PATZ1* RNAi fpkm**	***PATZ1* RNAi fpkm**
BMP4	0.804634	0.00765	0.119927	2.102038	1.936085	2.474329	3.034034	3.196468	4.94456

CDX2	0.327828	0.85761	1	0.016202	0	0.065979	0.030545	0.069312	0
GCM1	1.035888	0.656036	1	0	0.012704	0.013822	0.025596	0.029041	0
REST	0.052577	0.751318	0.920855	35.385	26.80231	25.26986	26.94994	31.91384	30.07908
FOXA1	2.234314	0.173625	1	0	0.055273	0.03007	0.278417	0	0.122665
FOXA2	1.242109	0.325449	0.692601	1.074382	0.626472	0.015852	2.113518	0.782665	1.034639
GATA2	0.630956	0.380896	0.735372	0.212336	0.278148	0.108084	0.340257	0.136252	0.440914
GATA4	0.336035	0.748349	1	0.194011	0.045383	0.123444	0.182877	0.025936	0.226609
GATA6	1.585369	0.086368	1	0.064437	0.048234	0.05248	0.315845	0.027565	0.133803
MSX1	0.683585	0.434641	1	0.153354	0.057396	0.37469	0.202375	0.229611	0.477657
AFP	1.095602	0.002635	0.065373	1.738013	4.481109	2.437796	6.407476	4.089255	8.060012
SOX17	4.582916	0.02746	0.241575	0.032059	0.014998	0	0.634604	0	0.449348
FGF5	2.542085	0.491889	1	0	0	0	0	0	0.042137
NES	0.264487	0.129477	0.495771	82.15404	92.66266	104.987	100.9394	100.5604	130.0021
NOG	0.665894	0.222141	0.603663	0.337314	0.241353	0.303	0.44888	0.318306	0.597424
NODAL	0.143419	0.846593	0.956202	36.38597	21.9506	5.851747	17.96558	17.20673	33.9512
MYL4	1.098721	0.506129	1	0.102571	0	0.104422	0.048343	0.109697	0.266235
TRDN	0.033277	0.860742	0.96057	56.62428	36.25596	46.71376	35.87455	56.26863	47.59599
ACTA1	0.153731	0.545014	0.826817	15.43611	11.83422	24.81559	14.22514	17.95333	24.50224
TNNC2	0.485981	0.096401	0.440144	3.376178	3.724782	4.257883	4.749898	6.628635	4.342379
PRSS1	0.993088	0.775964	1	0.041805	0	0	0	0	0.086808
HNF4A	3.278841	0.11317	1	0	0.013753	0	0.055422	0	0.091566
ALB	1.366455	0.511016	1	0	0.032524	0.035387	0.065531	0.037175	0.072179
AFP	1.095602	0.002635	0.065373	1.738013	4.481109	2.437796	6.407476	4.089255	8.060012

ASGR2	1.406329	0.141562	1	0.16451	0.092356	0.066991	0.217097	0.351876	0.273281
DLX2	0.887135	0.44097	1	0.017445	0.032645	0.106557	0.082218	0.05597	0.144895
ABCA4	0.705455	0.485729	1	0.22731	0.032721	0.017801	0.065928	0.205701	0.163387
TTR	0.992998	0.774354	1	0	0	0.045175	0	0	0.092144
KCNH5	0.923375	0.300711	1	0.090066	0.084272	0	0.162077	0.043783	0.119013
PRAME	1.831087	0.002871	0.068919	0.188108	0.176008	0.191502	0.531941	0.775966	0.641708
ARMC3	0.126596	0.870325	1	0.212396	0.124208	0.351371	0.200208	0.312333	0.220519
LRP6	0.525737	0.007134	0.11603	25.40734	17.28396	16.71239	21.25815	31.44477	31.0686
RSPO2	1.891199	0.024144	1	0.079536	0.049613	0.161942	0.349869	0.3686	0.330312
WNT7B	1.655497	0.110978	1	0.058415	0	0.118937	0.165187	0.124945	0.242594
SOX9	0.379437	0.10298	0.45128	2.504013	1.421856	1.984863	2.864813	2.177125	2.421164
TGFBR2	0.334027	0.096355	0.440144	2.457708	2.505201	2.12094	2.500776	3.350831	2.974165
LEF1	0.810954	0.220365	0.601483	0.411496	0.177704	0.644492	1.01446	0.236967	0.821601
EDA	0.257326	0.19596	0.577507	6.250274	6.051137	7.647685	6.430574	8.624454	8.43405
MC4R	1.865384	0.325205	1	0	0.020494	0.022298	0.041291	0.070273	0.04548
SRD5A2	3.556637	0.059257	1	0.008077	0	0	0.02284	0.008638	0.067086
IRS4	1.194032	0.06149	0.356606	1.424038	0.938962	0.428108	0.945929	2.361107	2.966918
SDC3	0.433718	0.013014	0.162794	29.15371	20.12384	23.50711	26.17889	36.60066	33.4861
CYP19A1	1.810069	0.214671	1	0.096507	0	0.032749	0.060646	0.137615	0.233794
PRKDC	0.373686	0.055412	0.342272	152.2854	138.8067	101.1004	132.4172	170.6403	197.9086
FSHR	2.304097	0.359918	1	0.025731	0	0	0.024254	0.110073	0
SHBG	0.737382	0.295434	0.670665	0.659387	0.370182	0.223761	0.870166	0.846233	0.319482
IRS1	0.338125	0.089183	0.424596	9.721706	6.922011	5.86938	7.569505	10.12967	10.20039

KLK3	2.14458	0.31294	1	0.064228	0	0	0	0.240413	0.033342
COL2A1	1.940539	5.11E-16	4.67E-12	4.101126	2.601251	3.512716	9.552933	11.4079	17.31846
NCF2	0.532856	0.683179	1	0.049625	0.18573	0.02526	0	0.079608	0.309135

DEA of cell cycle-regulatory genes upon *PATZ1* RNAi

Gene_ name	log2 fold change	pvalue	P adjust	Control RNAi fpkm	Control RNAi fpkm	Control RNAi fpkm	*PATZ1* RNAi fpkm	*PATZ1* RNAi fpkm	*PATZ1* RNAi fpkm
E2F1	0.539141	0.003424	0.07649	19.72294	16.89646	12.59694	22.32348	23.43498	24.55708
CCNE1	0.063459	0.712033	0.905624	42.34353	46.0585	35.5055	40.9349	43.43739	43.59805
CCNE2	0.547712	0.017612	0.190098	8.792892	6.811527	4.911712	8.476672	10.04489	10.93721
CDC25A	0.341169	0.099495	0.446434	47.11946	58.07373	33.97187	56.77533	55.72126	62.39492
CDC6	0.212673	0.319162	0.688397	87.01065	88.56911	49.60306	86.30194	81.53266	89.86381
CDKN1A	-1.98521	0.000884	0.031902	41.5576	44.52147	132.6466	11.71145	21.30591	21.29544
CDK3	-0.02907	0.954521	0.988175	0.829821	0.465864	1.621999	1.188944	0.780972	0.792631
MCM2	0.136806	0.518004	0.81339	101.1939	134.4376	76.57096	114.1367	106.6959	120.2164
MCM6	0.688812	0.000309	0.016687	107.4235	99.94689	66.1919	132.5049	151.8713	150.462
NPAT	0.408711	0.036398	0.278013	11.64572	10.1791	7.611144	11.05271	13.68485	13.74968
PCNA	0.038931	0.831637	0.952242	147.6079	206.6747	184.7833	193.5401	177.4166	178.0641
SLBP	-0.01985	0.898408	0.972809	45.53407	48.92276	48.55875	46.5386	45.27183	47.27544
BRCA1	0.192658	0.323196	0.69091	25.02739	15.45151	14.7756	20.45457	21.9012	19.2403
BRCA2	0.307462	0.180342	0.558724	6.730202	4.360727	3.091303	5.418372	5.9133	5.819068
CCNG2	-0.06973	0.704459	0.902478	14.635	9.654985	14.73552	11.33511	12.32383	12.63098

DHFR	0.525578	0.003375	0.075813	115.0111	100.4261	74.35662	124.0972	142.2198	144.2524
MSH2	0.308082	0.106223	0.457034	124.9456	105.5784	72.66757	113.4158	130.7612	125.0855
NASP	0.299057	0.059062	0.349918	626.1305	750.3659	721.2724	813.5725	873.4663	865.7558
RRM1-AS1	-0.8431	0.07761	0.396919	2.929506	4.365384	4.418306	2.556845	2.55283	1.351796
RRM2	0.511541	0.043132	0.300807	54.00779	86.13083	38.52594	91.04194	74.85872	88.35781
TYMS	-0.19558	0.258446	0.638769	90.07616	99.01085	126.6078	91.92094	87.89602	91.65753
BIRC5	-0.05656	0.710203	0.904935	136.7891	139.6883	158.3332	143.2523	141.0054	127.3286
BUB1	-0.25844	0.147807	0.522025	160.9528	140.6336	217.8586	149.5263	150.6081	125.552
BUB1B	0.094034	0.538212	0.824333	174.9506	157.2063	183.2272	156.0177	206.6867	178.6037
CCNB1	-0.37104	0.064016	0.364438	241.9	302.1704	404.274	270.9214	230.5174	221.4057
CDC20	-0.30656	0.047823	0.31771	208.6012	214.9159	253.8861	189.1677	177.2527	172.5489
CDC25B	-0.10907	0.57447	0.843649	18.3221	13.47843	23.32978	15.9249	18.61616	15.45008
CDC25C	-0.3981	0.033435	0.265683	26.41002	23.04076	35.3052	22.86528	19.90607	20.19706
CDKN2D	-0.17108	0.608743	0.860202	1.886035	1.512608	2.163878	1.439174	1.440755	1.958163
CENPA	-0.4845	0.027366	0.241196	28.02262	20.43761	39.84543	21.56013	22.41054	17.66308
CCNA2	-0.33358	0.023908	0.225783	83.64534	84.3303	97.02843	69.68544	70.80989	66.51274
CCNF	-0.16738	0.312828	0.684361	25.06378	28.71965	30.91447	24.8898	23.81921	25.69844
CENPF	-0.07588	0.589299	0.851949	231.878	177.794	205.7547	178.3716	197.3279	196.2842
TOP2A	-0.11914	0.443627	0.77055	454.7559	322.4182	457.8502	354.5155	402.8876	354.3603
CCNB2	-0.65583	0.000176	0.011753	96.89538	88.35166	129.2301	69.66505	66.93604	59.18243
CKS2	-0.35693	0.073711	0.389492	238.4747	243.1896	387.1712	239.6254	231.2664	195.5197

DEA of positive impact to flux in TCA cycle genes upon *PATZ1* RNAi

Gene_ name	log2 fold change	pvalue	P adjust	Control RNAi fpkm	Control RNAi fpkm	Control RNAi fpkm	*PATZ1* RNAi fpkm	*PATZ1* RNAi fpkm	*PATZ1* RNAi fpkm
ACSS2	0.254632	0.186746	0.567668	17.39254	19.02664	16.22763	18.74655	24.81434	18.59904
MPC2	0.295947	0.059338	0.350603	29.5099	30.02904	28.3944	35.62148	36.08754	34.67834
DLAT	0.2458	0.161048	0.537798	37.29966	48.03016	38.78671	46.15353	49.50855	50.27189
MPC1	0.043913	0.861738	0.961002	10.98455	16.18479	8.783365	12.57578	11.79266	12.54073
PDPR	0.172254	0.397964	0.744922	34.53427	31.47695	24.64213	24.2513	35.73588	40.77932
PDP2	0.069587	0.721294	0.90936	31.57535	24.26392	36.16654	24.08933	37.84989	32.8108
ACACB	0.120355	0.53406	0.822037	5.44645	3.770831	5.009562	4.610874	5.606188	4.911892
LDHB	-0.0184	0.928781	0.980652	148.1059	163.983	161.7544	203.1735	137.3244	119.7798
PDHX	-0.0951	0.53832	0.824333	50.79288	50.33043	50.77284	48.71296	48.45575	42.83201
DLD	-0.02141	0.886681	0.969181	84.29735	85.64521	88.65146	88.05877	84.63049	78.17829
PDHA2	-0.88997	0.703023	1	0.051567	0.024125	0.026249	0	0.05515	0
PDHB	-0.04143	0.794533	0.938516	82.05861	94.71953	90.04531	83.99465	91.67235	80.63811
PDP1	-0.75247	0.000168	0.011426	29.25932	17.25637	27.00645	11.55527	14.83972	16.15335
PDHA1	-0.19874	0.21669	0.600727	83.09941	86.5174	101.6997	83.96854	75.15357	73.46131
ACSS1	-0.43322	0.205029	0.587805	0.654458	0.728495	1.114271	0.606265	0.506841	0.702917

DEA of negative impact to flux in TCA cycle genes upon *PATZ1* RNAi

Gene_ name	log2 fold change	pvalue	P adjust	Control RNAi fpkm	Control RNAi fpkm	Control RNAi fpkm	*PATZ1* RNAi fpkm	*PATZ1* RNAi fpkm	*PATZ1* RNAi fpkm

LDHA	0.127211	0.485601	0.795027	355.5421	405.3888	292.3308	423.4305	350.1026	362.9646
LDHB	-0.0184	0.928781	0.980652	148.1059	163.983	161.7544	203.1735	137.3244	119.7798
LDHC	-0.27931	0.553094	0.831316	1.43373	1.023776	1.037081	1.173631	1.089472	0.548417
PDK2	0.011363	0.95191	0.98687	12.10655	12.00002	14.48274	12.25837	13.94651	12.08466
PDK1	0.084023	0.654309	0.880367	18.76309	15.06201	11.8248	14.16312	16.92917	16.45033
PDK3	0.170015	0.411391	0.753652	8.183989	5.989391	5.410377	5.857778	8.008019	7.756572
PDK4	-1.50482	0.17226	1	0.228445	0.07125	0.232566	0.047852	0.054292	0.07906
PCK2	-0.22751	0.247555	0.628104	101.1687	54.21313	60.77968	56.52818	62.30812	60.65518

DEA of TCA cycle regulatory genes upon *PATZ1* RNAi

Gene_ name	log2 fold change	pvalue	P adjust	Control RNAi fpkm	Control RNAi fpkm	Control RNAi fpkm	*PATZ1* RNAi fpkm	*PATZ1* RNAi fpkm	*PATZ1* RNAi fpkm
MFN1	0.34806	0.027004	0.239642	22.33029	18.75896	23.98056	24.71297	28.51328	28.05363
DNM1L	0.209228	0.159321	0.535717	73.26809	62.61406	66.40759	67.5673	84.87886	77.45653
OPA1	0.115679	0.427105	0.762335	45.02476	40.13277	42.76266	40.27933	50.46386	45.61987
MLYCD	0.051042	0.79629	0.938932	1.315202	1.046207	1.299677	1.292224	1.351588	1.071939
SUCLG2	0.05109	0.730627	0.9136	53.3841	46.80796	53.47063	49.28578	58.49698	48.69862
SDHAF3	0.01772	0.918348	0.978243	25.98409	24.31257	30.73831	26.11621	29.99924	24.55418
EME2	0.398454	0.056799	0.344926	18.08051	16.16553	19.74052	22.93799	16.73967	30.03515
CPT1C	0.169621	0.269639	0.646928	48.73174	42.68261	45.00898	51.0016	45.48457	54.0581
DLST	0.023184	0.870431	0.964078	135.1803	129.743	129.0024	132.2708	131.4914	130.1328
SUCLA2	0.009346	0.948408	0.98618	75.38271	63.34971	65.78341	64.10306	72.70075	65.35843

TFAM	0.188346	0.286961	0.662716	64.91555	59.51829	43.44015	61.34626	63.03518	63.96237
PDK1	0.084023	0.654309	0.880367	18.76309	15.06201	11.8248	14.16312	16.92917	16.45033
PPARGC1B	0.103793	0.707749	0.903977	5.388421	3.269822	2.503153	2.485356	4.498305	4.758034
GLUD1	-0.02266	0.875509	0.96607	144.1149	145.4532	145.835	138.9402	149.0986	134.373
PDHA2	-0.88997	0.703023	1	0.051567	0.024125	0.026249	0	0.05515	0
IDH3A	-0.17123	0.275602	0.652063	30.63859	25.86679	27.2476	23.86779	25.19673	23.94914
ACO1	-0.33165	0.024356	0.227643	18.95077	18.89419	20.69022	14.81255	16.01861	14.97759
OGDHL	-0.68759	0.000218	0.013517	15.13728	11.43809	18.35995	9.098613	9.660295	8.525667
MDH1	-0.36954	0.015226	0.175661	418.4014	399.3369	476.4085	351.7067	336.7339	295.963
SUCLG1	-0.23818	0.165594	0.541964	51.94688	50.57677	54.72272	50.58723	39.27524	41.03053
ME1	-0.12076	0.58769	0.851202	2.728438	2.750903	3.208482	3.201708	2.370116	2.266226
DLD	-0.02141	0.886681	0.969181	84.29735	85.64521	88.65146	88.05877	84.63049	78.17829
SDHAF1	-0.1829	0.348606	0.71178	11.89753	13.4278	12.45432	11.75334	11.21442	9.875047
OGDH	-0.11628	0.493878	0.799335	35.06814	43.11312	44.22409	37.80203	35.75177	38.03329
HADHA	-0.10976	0.495213	0.800006	76.47963	87.71127	93.20169	81.98527	79.99239	73.43594
IDH3G	-0.03579	0.824379	0.949569	28.39153	29.53861	30.71689	28.67149	28.2724	28.24456
ECH1	-0.28029	0.132297	0.498824	84.42074	111.6696	117.9508	90.02199	84.69252	81.05618
ACO2	-0.18018	0.256558	0.637919	78.16982	90.10362	91.09537	74.51964	76.6036	74.96998
IDH3B	-0.23833	0.157181	0.532652	100.8566	119.8482	119.4466	102.9913	90.32537	91.33363
SDHA	-0.36064	0.037155	0.280441	64.64509	67.70356	90.41166	58.72369	56.32981	55.6091
SDHB	-0.32993	0.07873	0.399401	83.91851	98.06248	130.1387	77.89121	85.85367	81.23405
SDHC	-0.09869	0.52593	0.81724	87.31638	90.15275	107.6002	87.17654	93.12603	81.91518
MDH2	-0.1442	0.40464	0.749207	201.4408	245.9469	282.6275	209.5115	232.7253	210.7093

PDHB	-0.04143	0.794533	0.938516	82.05861	94.71953	90.04531	83.99465	91.67235	80.63811
PRPS1	0.061836	0.682474	0.893426	95.15554	102.6393	100.1144	98.12467	108.8187	100.0304
NRF1	-0.01032	0.959073	0.988589	8.796497	10.20073	7.256247	9.364916	8.623622	7.788878
SDHAF2	-0.13194	0.591159	0.852882	11.38836	17.68941	13.88508	15.02027	10.39831	13.45961
MFN2	0.020413	0.909186	0.976217	47.817	63.12295	50.12621	52.52689	54.64059	54.88392

DEA of glycolysis genes upon *PATZ1* RNAi

**Gene_ name**	**log2 fold change**	**pvalue**	**P adjust**	**Control RNAi fpkm**	**Control RNAi fpkm**	**Control RNAi fpkm**	***PATZ1* RNAi fpkm**	***PATZ1* RNAi fpkm**	***PATZ1* RNAi fpkm**
PGAM2	0.178708	0.684696	0.894264	0.876105	1.844428	2.631135	2.518776	1.873935	1.591817
ENO3	-0.19634	0.262905	0.641724	316.4177	403.8881	354.3883	340.5817	295.0742	292.3028
ENO1	-0.06624	0.699765	0.900304	1506.315	1917.775	1704.853	1753.272	1551.808	1541.786
TPI1	-0.04494	0.790259	0.93675	617.3205	744.5859	710.8136	736.2428	638.4199	609.8123
BPGM	-0.0457	0.80947	0.944058	12.25977	13.72079	15.0256	14.58553	13.23875	11.31982
LDHB	-0.0184	0.928781	0.980652	148.1059	163.983	161.7544	203.1735	137.3244	119.7798
GAPDHS	-0.34888	0.34769	0.711151	1.897237	2.039581	2.260225	1.560061	2.029038	1.215405
PGK1	-0.08814	0.551826	0.830594	245.3528	266.8444	254.121	237.237	242.1724	232.0602
ENO2	-0.42664	0.04815	0.319533	94.48153	70.45816	138.8856	83.3989	75.89313	61.30518
PFKL	-0.13669	0.374857	0.730362	42.77763	38.1305	50.48427	38.9933	38.56042	39.70895
GCK	-0.14351	0.579483	0.846445	3.170924	3.67237	4.988928	3.469698	4.268652	2.831744
PFKM	-0.01437	0.92795	0.980652	76.15842	79.07571	95.8198	80.38708	87.14947	77.31502
PFKP	-0.28466	0.112663	0.469423	108.5718	115.9509	152.2301	86.50482	108.2832	110.1769

PKM	-0.12078	0.483637	0.793876	467.8639	563.2172	653.6536	463.554	551.0072	517.2096
FBP2	0.012253	0.987812	1	0.316438	0.161499	0.205003	0.244046	0.215359	0.209071
HK2	0.151865	0.435317	0.766644	31.65653	25.3663	17.93207	25.34117	27.68864	28.74984
LDHA	0.127211	0.485601	0.795027	355.5421	405.3888	292.3308	423.4305	350.1026	362.9646
PGAM1	0.114742	0.472295	0.788496	329.1176	369.6191	301.0144	349.5763	359.7986	360.7099
GAPDH	0.138462	0.451429	0.775888	2437.129	3282.436	2389.33	3064.143	2889.552	2903.816
ALDOC	0.037486	0.831022	0.952011	42.10786	52.0499	45.50327	43.75889	52.10916	46.18225
HK1	0.116085	0.457168	0.777905	141.4741	138.5944	152.3588	129.8736	175.0673	157.1787

DEA of energy genes upon *PATZ1* RNAi

**Gene_ name**	**log2 fold change**	**pvalue**	**P adjust**	**Control RNAi fpkm**	**Control RNAi fpkm**	**Control RNAi fpkm**	***PATZ1* RNAi fpkm**	***PATZ1* RNAi fpkm**	***PATZ1* RNAi fpkm**
ALDOA	0.103171	0.610865	0.861218	11.50619	13.62623	11.64383	12.10855	12.01165	14.94169
PPA1	0.185524	0.221636	0.602956	116.4844	123.7459	112.4419	133.5031	136.5831	125.6555
PDK3	0.170015	0.411391	0.753652	8.183989	5.989391	5.410377	5.857778	8.008019	7.756572
PDK1	0.084023	0.654309	0.880367	18.76309	15.06201	11.8248	14.16312	16.92917	16.45033
IDH3A	-0.17123	0.275602	0.652063	30.63859	25.86679	27.2476	23.86779	25.19673	23.94914
NDUFB8	-0.12993	0.480945	0.792274	37.06285	33.11251	39.07035	38.14781	31.58221	28.11541
COX8A	-0.46617	0.006507	0.110451	335.056	352.5082	379.6959	298.4491	236.9348	224.5585
COX5B	-0.28083	0.074372	0.390194	247.5576	251.8774	279.8865	233.0863	200.5649	197.2178
NDUFS7	-0.45835	0.004903	0.094953	39.82961	40.51183	49.96155	32.14005	31.02651	30.1206
COX7A2	-0.39319	0.027796	0.243109	224.8568	233.3638	311.1027	215.3204	185.5331	174.856

NDUFB5	-0.26685	0.096965	0.440953	89.94874	86.92975	102.2501	83.44877	77.00857	67.51334
NDUFS4	-0.03437	0.820488	0.948148	81.43896	78.89347	90.29543	79.1962	82.47871	79.07429
SDHB	-0.32993	0.07873	0.399401	83.91851	98.06248	130.1387	77.89121	85.85367	81.23405
MDH2	-0.1442	0.40464	0.749207	201.4408	245.9469	282.6275	209.5115	232.7253	210.7093
UQCRH	-0.21978	0.178362	0.556989	458.3365	535.6312	563.1568	470.1301	429.7293	419.6686
CYC1	-0.24549	0.144732	0.517555	119.6178	145.8477	146.819	121.5762	111.2682	110.7207
PGK1	-0.08814	0.551826	0.830594	245.3528	266.8444	254.121	237.237	242.1724	232.0602
NDUFB10P1	1.956355	0.627805	1	0	0	0	0	0	0.149623
UQCRC1	-0.24423	0.162554	0.5397	212.7725	276.8086	259.8443	221.9816	197.9412	206.1026
PDHB	-0.04143	0.794533	0.938516	82.05861	94.71953	90.04531	83.99465	91.67235	80.63811
TPI1	-0.04494	0.790259	0.93675	617.3205	744.5859	710.8136	736.2428	638.4199	609.8123
BPGM	-0.0457	0.80947	0.944058	12.25977	13.72079	15.0256	14.58553	13.23875	11.31982

DEA of cell ETC genes upon *PATZ1* RNAi

**Gene_ name**	**log2 fold change**	**pvalue**	**P adjust**	**Control RNAi fpkm**	**Control RNAi fpkm**	**Control RNAi fpkm**	***PATZ1* RNAi fpkm**	***PATZ1* RNAi fpkm**	***PATZ1* RNAi fpkm**
MT-ND1	0.510751	0.003367	0.075719	1480.595	1649.311	1287.791	2269.934	1828.168	2113.552
NDUFS7	-0.45835	0.004903	0.094953	39.82961	40.51183	49.96155	32.14005	31.02651	30.1206
NDUFB1	0.399209	0.014036	0.168867	61.45423	67.1847	71.13587	89.67873	85.59172	84.42743
NDUFB7	-0.43118	0.017845	0.191599	155.1226	198.2306	204.2879	150.6941	130.295	127.6673
MT-ND2	0.416704	0.024496	0.22807	1609.655	1955.567	1766.957	2803.024	1988.38	2232.486
NDUFS8	-0.3581	0.029133	0.249045	100.0773	112.2223	128.1178	87.80478	87.2918	86.80505

SDHA	-0.36064	0.037155	0.280441	64.64509	67.70356	90.41166	58.72369	56.32981	55.6091
SDHB	-0.32993	0.07873	0.399401	83.91851	98.06248	130.1387	77.89121	85.85367	81.23405
SDHC	-0.09869	0.52593	0.81724	87.31638	90.15275	107.6002	87.17654	93.12603	81.91518
SDHD	-0.0828	0.652596	0.87991	73.53314	88.49734	86.2232	89.41164	76.01033	65.98616
UQCR11	-0.48226	0.005753	0.10322	54.33858	62.51849	62.53912	46.14351	40.94747	39.58579
UQCRC2	-0.27828	0.073748	0.389492	174.0158	174.3389	218.2233	153.5474	154.8137	151.2092
UQCRFS1	-0.28209	0.082052	0.407268	43.0399	48.41844	53.75865	41.47632	38.95902	37.31676
CYC1	-0.24549	0.144732	0.517555	119.6178	145.8477	146.819	121.5762	111.2682	110.7207
UQCRC1	-0.24423	0.162554	0.5397	212.7725	276.8086	259.8443	221.9816	197.9412	206.1026
UQCRH	-0.21978	0.178362	0.556989	458.3365	535.6312	563.1568	470.1301	429.7293	419.6686
COX17	-0.51945	0.002409	0.06154	78.87693	83.12334	102.7894	62.34198	61.52805	57.97267
COX8A	-0.46617	0.006507	0.110451	335.056	352.5082	379.6959	298.4491	236.9348	224.5585
COX7A2	-0.39319	0.027796	0.243109	224.8568	233.3638	311.1027	215.3204	185.5331	174.856
COX6C	-0.33474	0.049247	0.323062	227.8519	218.0146	284.8895	215.2228	181.5573	171.8298
COX14	-0.33976	0.065534	0.369194	28.82222	27.5397	28.94644	24.13208	23.51926	18.62352
ATP1A2	0.999501	9.10E-07	0.000302	39.65378	27.93362	36.51424	46.21723	84.55633	73.38095
ATP6V0C	-0.68497	0.001141	0.03808	24.15879	25.41477	21.40228	17.52684	11.76131	14.12335
ATP6V1E1	-0.51266	0.001294	0.041454	117.6403	114.6455	138.6516	92.70674	83.25839	79.48029
ATP8B3	-1.13961	0.001382	0.043273	2.116722	1.661111	5.018852	1.634819	1.343657	0.907426
ATP6V1C2	-0.76965	0.00346	0.077057	7.677211	8.945299	10.19975	7.373186	3.847079	4.210996
ATP6V0E2-AS1	-1.06388	0.00553	0.101203	1.044775	0.811877	1.099679	0.433988	0.435579	0.514788
ATP1A3	0.705181	0.007126	0.11603	1.861722	2.274795	2.363562	2.807816	4.169504	3.524737
ATP2A1	-0.62047	0.017403	0.189266	5.877121	3.924435	8.17082	3.441056	4.264106	3.709514

ATP8A2	0.492267	0.020063	0.203733	2.354157	1.724258	1.650137	2.582841	2.847889	2.464222
ATP6V0A2	0.33623	0.040452	0.291433	25.02739	20.9985	18.97193	23.86026	27.9873	28.80033
DNM1L	0.209228	0.159321	0.535717	73.26809	62.61406	66.40759	67.5673	84.87886	77.45653
MFN1	0.34806	0.027004	0.239642	22.33029	18.75896	23.98056	24.71297	28.51328	28.05363
MFN2	0.020413	0.909186	0.976217	47.817	63.12295	50.12621	52.52689	54.64059	54.88392
OPA1	0.115679	0.427105	0.762335	45.02476	40.13277	42.76266	40.27933	50.46386	45.61987

DEA of cell mitochondria biogenesis genes upon *PATZ1* RNAi

**Gene_ name**	**log2 fold change**	**pvalue**	**P adjust**	**Control RNAi fpkm**	**Control RNAi fpkm**	**Control RNAi fpkm**	***PATZ1* RNAi fpkm**	***PATZ1* RNAi fpkm**	***PATZ1* RNAi fpkm**
NRF1	-0.01032	0.959073	0.988589	8.796497	10.20073	7.256247	9.364916	8.623622	7.788878
MFN2	0.020413	0.909186	0.976217	47.817	63.12295	50.12621	52.52689	54.64059	54.88392
MFN1	0.34806	0.027004	0.239642	22.33029	18.75896	23.98056	24.71297	28.51328	28.05363
DNM1L	0.209228	0.159321	0.535717	73.26809	62.61406	66.40759	67.5673	84.87886	77.45653
OPA1	0.115679	0.427105	0.762335	45.02476	40.13277	42.76266	40.27933	50.46386	45.61987
PPARGC1B	0.103793	0.707749	0.903977	5.388421	3.269822	2.503153	2.485356	4.498305	4.758034
TFAM	0.188346	0.286961	0.662716	64.91555	59.51829	43.44015	61.34626	63.03518	63.96237

Supplementary Table 6. Calculation equations of ATP production from Seahorse assay.

Calculated values	Equations
OCR_{mito}	$= (OCR_{total} - OCR_{rot/myc})$
OCR_{couple}	$= \left(OCR_{total} - OCR_{oligo}\right) \times hyperpolarization\ correction\ factor$
PPP_{total}	$= (\frac{ECR_{total}}{buffering\ power})$
PPP_{resp}	$= (OCR_{mito} \times K_{PPRresp})$
PPP_{glyc}	$= (PPR_{total} - PPR_{resp})$
$Glycolytic\ ATP\ J(_{ATPglyc})$	$= \left(PPR_{glyc} \times \frac{ATP}{lactate}\right) + (OCR_{mito} \times 2\ \times \frac{ATP}{[O]_{glyc}})$
$Oxidative\ ATP\ J(_{ATPox})$	$= \left(OCR_{Couple} \times 2 \times \frac{ATP}{[O]_{oxphos}}\right) + (OCR_{mito} \times 2 \times \frac{ATP}{[O]_{TCA}})$

Parameters	Values
hyperpolarization correction factor	0.908
buffering powder (mpH/pmol H^+)	0.045
$\frac{ATP}{lactate}$	1.000
$\frac{ATP}{[O]_{glyc}}$	0.167
$\frac{ATP}{[O]_{oxphos}}$	2.486
$\frac{ATP}{[O]_{TCA}}$	0.121

Supplementary Table 7. Quantification of metabolites by LC/MS/MS

**Quantification of metabolites by LC/MS/MS**							
**Metabolites**	**Class**	**HMDB**	**KEGG**	**Uni FDR**	FC	Log2FC	OPLSDA VIP
Glutamic acid	Amino acids	HMDB0000148	C00025	1.0E-4	1.546	0.6286	1.3633
Malic acid	Organic acids	HMDB0000156	C00149	0.0046	1.4843	0.5698	1.2566
Aspartic acid	Amino acids	HMDB0000191	C00049	0.0114	1.1999	0.2629	1.0457
Lactic acid	Organic acids	HMDB0000190	C00186	0.026	1.2872	0.3643	1.0922
Serine	Amino acids	HMDB0000187	C00065	0.0052	1.9245	0.9445	1.1881
Glutamine	Amino acids	HMDB0000641	C00064	<0.0001	3.4987	1.8068	1.3752
Glucose	Carbohydrates	HMDB0000122	C00221	0.0087	4.957	2.3095	1.2481
Fructose	Carbohydrates	HMDB0000660	C02336	<0.0001	5.0138	2.3259	1.3179
3-Phosphoglyceric acid	Carbohydrates	HMDB0000807	C00597	0.039	1.9587	0.9699	1.0235
Citric acid	Organic acids	HMDB0000094	C00158	0.0022	2.6074	1.3826	1.0625
Isocitric acid	Organic acids	HMDB0000193	C00311	0.0131	1.4121	0.4978	1.2048
Oxoglutaric acid	Organic acids	HMDB0000208	C00026	3.0E-4	2.1531	1.1064	1.0366
Glucose-1-phosphate	Carbohydrates	HMDB0001586	C00103	9.0E-4	3.1027	1.6335	1.1634
Fructose-1-phoshate	Carbohydrates	HMDB0001076	C01094	0.0035s	2.5133	1.3296	1.0644
